# Mapping QTL for summer dormancy related traits in tall fescue (*Festuca arundinacea* Schreb.)

**DOI:** 10.1038/s41598-020-71488-8

**Published:** 2020-09-03

**Authors:** Shyamal K. Talukder, Suresh Bhamidimarri, Konstantin Chekhovskiy, Malay C. Saha

**Affiliations:** 1 Noble Research Institute, LLC., 2510 Sam Noble Parkway, Ardmore, OK 73401 USA; 2 California Cooperative Rice Research Foundation, Rice Expt. Station, 955 Butte City Highway, Biggs, CA USA; 3 Corteva Agriscience, 1040 Settler Rd., Connell, WA USA

**Keywords:** Genetics, Plant sciences

## Abstract

Summer dormancy is an important stress avoidance mechanism of cool season perennial grasses to persist well under harsh summer conditions. QTL associated with summer-dormancy related traits in tall fescue has significant breeding implications. An F_1_ pseudo testcross population was developed by crossing a Mediterranean (103-2) to a Continental parent (R43-64). The population was genotyped using 2,000 SSR and DArT markers. Phenotyping was done in growth chambers and in two Oklahoma, USA locations. Total length of R43-64 and 103-2 maps were 1,956 cM and 1,535 cM, respectively. Seventy-seven QTL were identified in the male and 46 in the female parent maps. The phenotypic variability explained by the QTL ranged between 9.91 and 32.67%. Among all the QTL, five summer dormancy related putative QTL were identified in R43-64 linkage groups (LGs) 4, 5, 12, 20 and 22 and two in 103-2 LGs 5 and 17. All the putative summer dormant QTL regions in male map showed pleiotropic responses and epistatic interactions with other summer dormant and stress responsive QTL regions for plant height, new leaf and dry biomass weight. The flanking markers related to the QTL reported in this study will be useful to improve tall fescue persistence in dry areas through marker-assisted breeding.

## Introduction

Summer dormancy is a phenomenon, which is endogenously controlled and coupled with series of processes including growth reduction, cessation and/or senescence under non-limiting moisture conditions during summer^1^. It is an important drought avoidance mechanism of cool season perennial grasses in the Mediterranean climates to survive under harsh summer conditions^2^. Summer dormancy had been reported in many grass species, i.e., *Poa secunda* J. Presl [syn. *Poa scabrella* (Thurb.) Benth. ex Vasey]^3^, *Poa bulbosa* L.^4^, *Hordeum bulbosum* L.^5^, orchardgrass (*Dactylis glomerata* L.)^6^, hardinggrass (*Phalaris aquatica* L.)^7^ and tall fescue [*Festuca arundinacea* (Schreb.) S.J. Darbysh.]^8^.

Tall fescue is an important perennial, cool-season forage and turf grass in the United States^9^ belongs to the genus ‘*Festuca*’ under Poaceae family^10,11^. It is a cross-pollinated allohexaploid grass. Tall fescue has three distinct morphotypes, and those mainly used as forage belong to the Continental and Mediterranean groups. These two morphotypes might have differences in genomic constitution^12–14^. Sleper and West^15^ reported that the genomic constitution of Continental tall fescue is PPG1G1G2G2, while that of the Mediterranean fescue is still unresolved^9,11,14,16,17^. Continental morphotype is summer active and winter hardy. Mediterranean morphotype exhibits incomplete summer dormancy, greater growth during fall but lacks winter hardiness^18,19^. In the southern great plains, the persistence and productivity of cool season perennial grasses are significantly affected by hot and dry summer^20,21^. Thus, understanding and utilization of summer dormancy mechanism in tall fescue could help in developing suitable cultivars for the region.

The induction and exhibition of summer dormancy are controlled by cold followed by high temperature, long day and drought stresses. Ofir and Kigel^22^ found enhanced summer dormancy in *P. bulbosa* under the conditions of prior exposure to low temperature and short day followed by long day. Norton et al.^1^ reported the requirement of long day and dry summer for dormancy in tall fescue. The requirement of plant exposure under multi stress condition makes phenotyping for summer dormancy difficult. Furthermore, confounded with the response of drought and high temperature exhibited by the plants makes it harder to demonstrate unequivocal occurrence of summer dormancy. Particularly, under severe water stress condition, temperate perennial grasses manifests gradual decrease in leaf elongation, leading eventually to cessation and ultimately progressive senescence of all mature leaves^23^. However, demonstration of all the progressive effects under particular circumstances is time consuming. Considering all the complexity, phenotyping summer dormancy for tall fescue breeding might not be highly efficient. Marker-assisted selection (MAS) is an appealing method for selecting complex phenomenon like summer dormancy. Identification and use of marker flanked to quantitative trait loci (QTL) associated with summer dormancy related traits will pose significant advantage over phenotypic selection to the breeders.

Dierking et al.^24^ reported genetic linkage map of an F_1_ pseudo-testcross population between a Mediterranean and a Continental tall fescue genotype using SSR and Diversity Array Technology (DArT) markers. In this study, we have incorporated additional SSR marker and rebuilt the genetic linkage maps. We also conducted both growth chamber and field studies for phenotyping summer dormancy related traits in the population. Using both the phenotypic and genotypic data, QTL and molecular marker that are associated with summer dormancy related traits in tall fescue have been identified and reported in this study. The identified marker could be used for MAS to enhance tall fescue breeding program for summer dormancy.

## Results and discussion

### Phenotypic variability and performance in the growth chamber study

Long drought spell is required during harsh summer to visualize summer dormancy in tall fescue^1^. Thus, phenotyping for summer dormancy is difficult and critical, due to the confounding response of drought, heat and other stresses with summer dormancy in plants. Bhamidimarri et al.^8^ suggested a procedure for in vitro phenotyping of summer dormancy in tall fescue. The growth differences between the optimum (short day and cooler temperature) and the summer dormant conditions (long day and high temperature) might be useful to identify summer dormancy in tall fescue.

**Figure 1:**
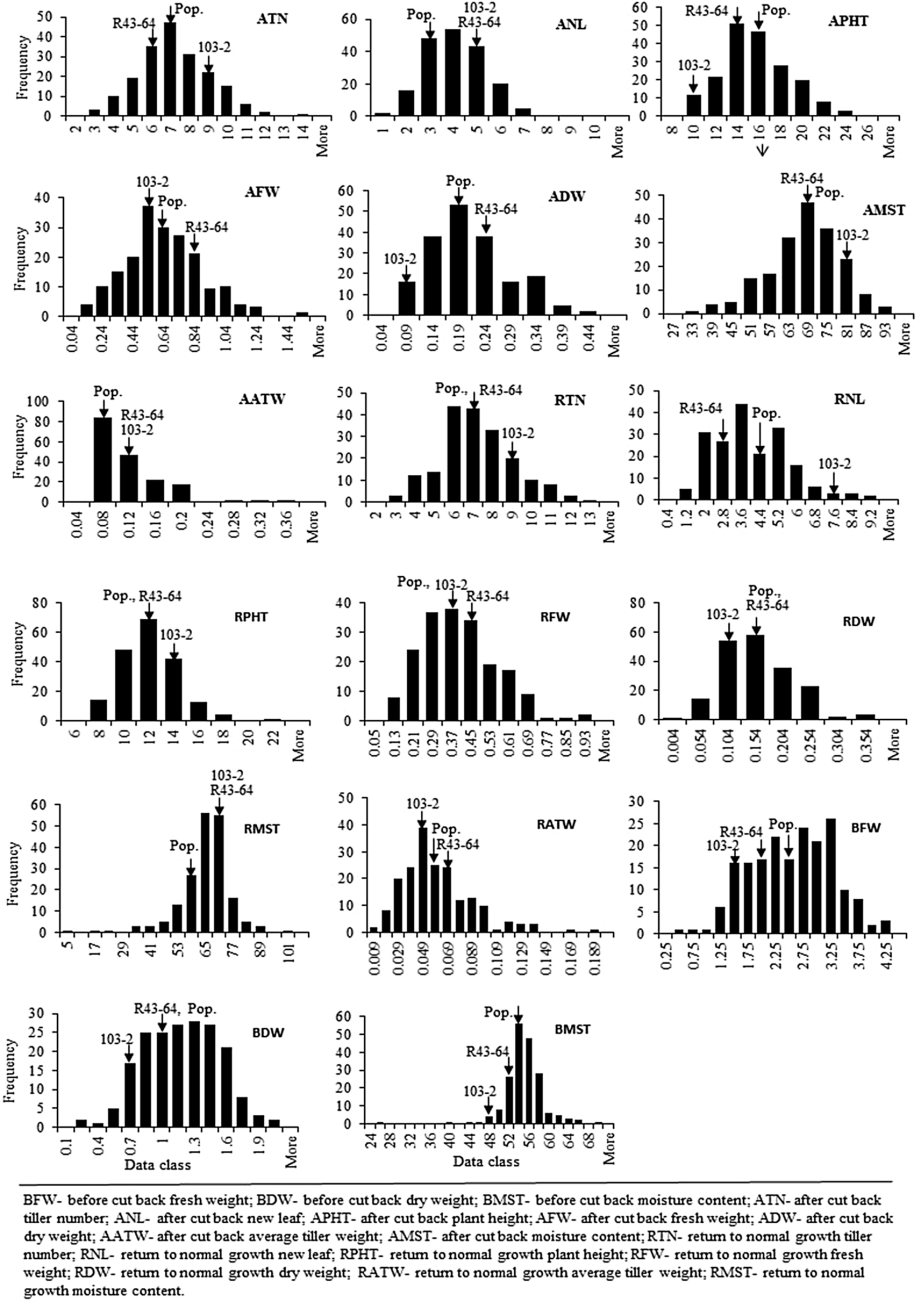
Distributions of trait values under summer dormant growing condition in the growth chamber study. X-axis represents the trait value and Y-axis the number of genotypes. Traits name are written upright corner of the distribution.

Significant variability among the genotypes for before cut back fresh weight (BFW), before cut back dry weight (BDW), after cut back tiller number (ATN), after cut back new leaf (ANL), after cut back plant height (APHT), after cut back dry weight (ADW), return to normal growth new leaf (RNL), return to normal growth plant height (RPHT), return to normal growth dry weight (RDW), and return to normal growth average tiller weight (RATW) traits was observed in growth chamber. Treatment effect was significant for all the measured traits except after cut back average tiller weight (AATW), return to normal growth tiller number (RTN) and return to normal growth fresh weight (RFW), while genotype*treatment was non-significant for all the measured traits (Supplementary Table S1). R43-64 and 103-2 parents showed considerable variability for 11 traits under optimum growing condition and for 14 traits under summer dormant condition. All the traits were normally distributed showing transgressive segregation except AATW (Fig. 1 and Supplementary Fig. S1). R43-64 performance, when compared between optimum and summer dormant conditions, was better for RNL, RFW, RDW, RATW, BFW and before cut back moisture content (BMST); worse for after cut back fresh weight (AFW), ADW, after cut back moisture content (AMST), AATW, return to normal growth moisture content (RMST) and BDW; and similar for ATN, ANL, APHT, RTN and RPHT. The 103-2 performed better for ATN, APHT, AFW, ADW, RFW, RDW, RATW, BFW, BDW and BMST and worse for ANL, AMST, AATW, RTN, RNL, RPHT and RMST under optimum condition compared to dormant condition.

Variability of phenotypic responses in mapping population is dependent on recombination events occurred during crossing. However, crossing incompatibility and hybrid sterility between the two morphotypes was reported earlier^14,25^. As a result, distorted phenotypic segregation in the population may not be unlikely due to potential uneven crossovers. Polygenic traits in a biparental population usually get re-assorted during recombination and a range of phenotypic value is produced. Thus, transgressive segregation in a population is highly likely which is useful for QTL mapping^26^. In summer dormancy related traits, it was reported in *D. glomerata*^27,28^. In this study, we find that summer dormancy is not only imbedded in the 103-2, but the Continental parent also contributes alleles.

The reduction of tiller number (TN), fresh biomass weight (FW) and dry biomass weight (DW) of 103-2 under summer dormant condition and no reduction of those traits in R43-64 was in agreement with the results of Bhamidimarri et al.^8^. In addition, the plant height of R43-64 under summer dormant conditions remained consistent, while it was significantly reduced in the 103-2. Thus, the phenotyping condition provided in this study was suitable to exhibit summer dormancy responses by the plants. The consistency of those above mentioned traits in the parental genotypes also support the earlier reports of Volaire and Norton^23^ and Norton et al.^1^, where they described that non dormant cocksfoot and tall fescue populations continued their active growth during wet summer while dormant ones showed reduced growth and performances.

The correlation analysis revealed significant positive, as well as negative correlations among the traits (Supplementary Table S2). Significant positive and negative correlations (r ≥ 0.4) were found in 15 and 3 traits combinations, respectively. Traits related to vegetative growth have a tendency to show correlation with each other during adaptation to new environment^29^. The genetic reasons behind trait correlation are pleiotropy and linkage disequilibrium (LD)^30^. The negative correlation observed between moisture content in summer dormancy condition and fresh weight of return to normal growing condition might be attributed to pleiotropy rather than LD. The dormant plants had growth cessation under summer dormant condition and had low moisture, while returned to the normal growth condition enhanced the growth to produce high fresh and average tiller weight. This in general might be true as pleiotropy is the predominant cause of trait association over linkage^31^. A negative correlation between summer dormancy and vegetative productivity was observed by Shaimi et al.^28^ and Kallida et al.^27^, however they described the correlation as environmental adaptation rather than genetic control.

### Phenotypic variability and performance in the field study

**Figure 2:**
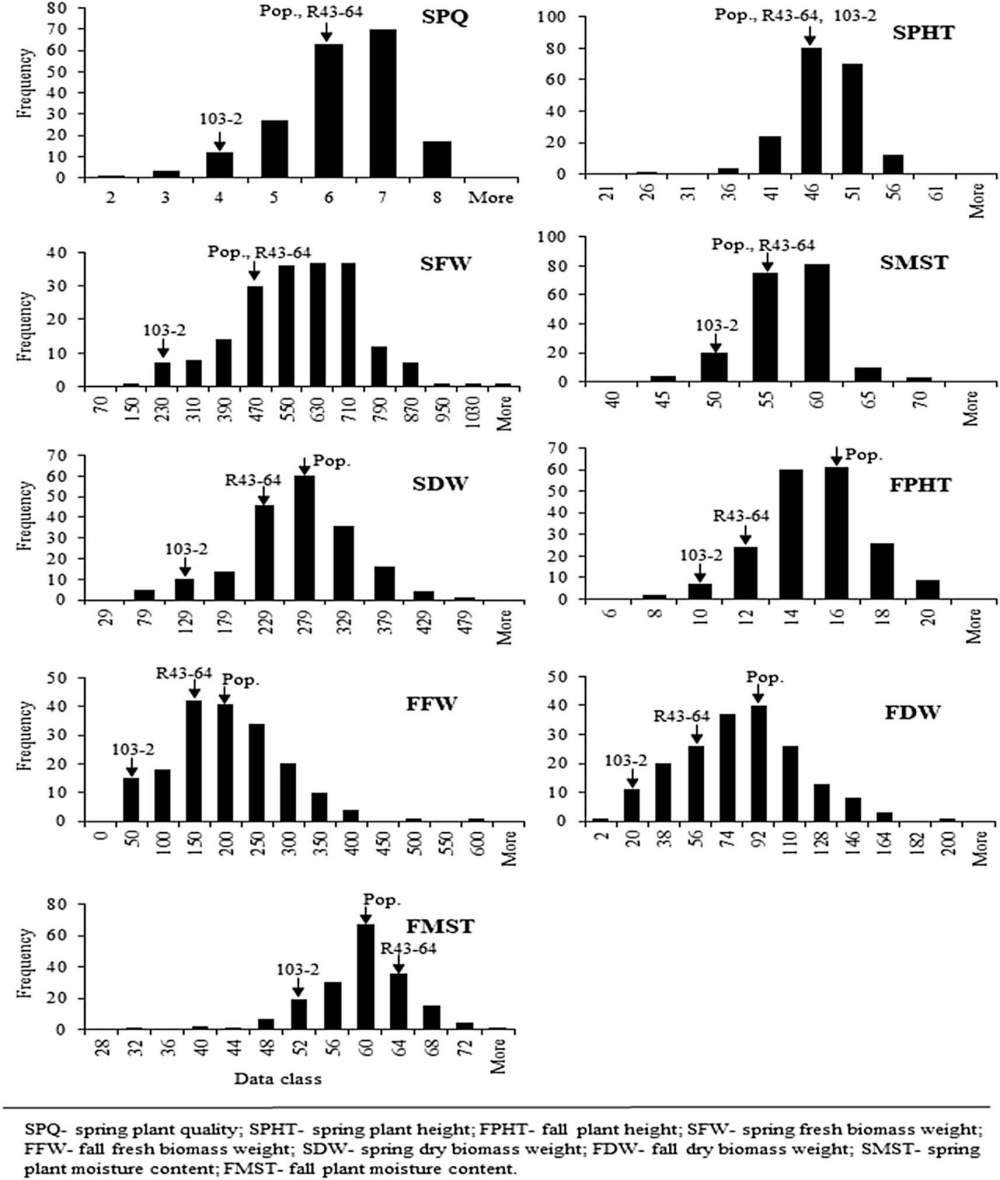
Distributions of trait values obtained from field experiments. X-axis represents the trait value and Y-axis as number of genotypes. Traits name are written upright corner of the distribution.

All the traits in the field experiments showed normal distribution (Fig. 2). Significant variability was found among the genotypes for spring plant quality (SPQ), spring fresh biomass weight (SFW), spring dry biomass weight (SDW), spring plant moisture content (SMST), fall plant height (FPHT), fall fresh biomass weight (FFW) and fall dry biomass weight (FDW), but not for spring plant height (SPHT) and fall plant moisture content (FMST) (Supplementary Table S1).

Location effect was significant in SPQ, FPHT, FDW and FMST and genotype*location effect was significant in SMST, FPHT and FDW traits. R43-64 showed superiority over 103-2 in SPQ (6/3), SFW (446.54/181.72g), SDW (198.8/83.8g), FFW (123.36/27.40g), FDW (44.0/14.5g) and FMST (61.89/50.79%), meanwhile no clear difference was found in SPHT (107.09/110.70 cm), SMST (54.72/53.91%) and FPHT (26.44/22.86 cm).

The overall performance of the population showed clear differences between fall and spring data. The fall harvest was done during the first week of October, thus the plants experienced harsh dry summer, which might have provided the conjugating effect of heat, drought, as well as summer dormancy. The overall performance of the population and parents during fall harvest was lower compared to the spring harvest, because growing condition before spring was merely optimum for the plants. It was notable that the fall fresh and dry weight of summer dormant parent (103-2) reduced to 15% and 17% of the spring harvest, while Continental parent reduced to 27% and 22%, respectively. High reduction of 103-2 biomass weight might be due to the cumulative effect of summer stress and summer dormancy. Plant moisture content reduced in summer dormant parent but not in Continental parent in between the two harvests. Continental parent could recover from the summer drought in terms of plant water content prior to fall harvest but summer dormant parent could not. This might be due to the sustained dormancy effect in summer dormant parent, because breaking dormancy takes longer time than recovery from simple drought stress.

### Linkage maps

The Continental parent (R43-64) map comprised of 817 markers (571 SSR and 246 DArTFest). Twenty-two LGs covered a total size of 1956.30 cM (Supplementary Fig. S2). The average LG size was 88.92 cM with a marker interval of 2.39 cM. LGs 8 and 22 consisted two groups each, decided by comparing the distribution of homologous and homeologous marker in both parental groups, as well as earlier reported groups of Dierking et al.^24^ The Mediterranean parental (103-2) maps accommodated 395 markers (211 SSR and 184 DArTFest). Twenty-three LGs were formed covering total size of 1534.56 cM (Supplementary Fig. S3). The average linkage group size was 69.75 cM with a marker interval of 4.32 cM. LGs 2, 4, 6, 12 and 19 comprised of two groups each and LG 14 comprised of three groups.

Based on the common marker, LGs of each parent were aligned to Dierking et al.^24^ maps. We added 223 additional markers in R43-64 and 187 markers in the 103-2 parental maps. Total length along of the LGs increased by 379 and 276 cM for R43-64 and 103-2 maps, respectively. We have included distorted marker during LG construction. The distorted markers made minor positional displacements in few groups. After QTL mapping, we again carefully checked the marker position and additive effects and decided to continue with the LGs including both distorted and non-distorted marker, because we did not find marker that had significant unwanted positional effect on the neighboring marker. Inclusion of even highly distorted marker in LGs can be beneficial. Segregation distortion would not produce false positive QTL, nor would it influence on positional effect of the QTL. Thus, distortion effect can be ignored in a large mapping population^32–34^. Consideration of segregation distortion as a random effect in a large population might be beneficial to QTL mapping^35^. Wang et al.^36^ supported that meticulous insertion of distorted marker enhanced QTL mapping by increasing marker coverage in the genome. Otherwise, QTL detection will be deprived due to fewer marker in the target region.

### QTL in growth chamber experiment

Total of 27 QTL were identified in 12 LGs of male parental map. Highest number of QTL (5) was found in LGs 6 and 22 followed by LG 12 (3). QTL identified in the male parental map had LOD values ranging between 2.18 and 4.79 and phenotypic variability explained (PVE) between 10.03 and 22.32%. QTL for all the traits except ATN were identified in the male LGs (Table 1). Total of 24 QTL were identified in 13 LGs of female parental map for all the measured traits. Highest number of QTL (4) were found in LG 14c followed by LG 23 (3). The LOD and PVE range of the QTL in female parental LGs were between 2.18–4.67 and 9.91–21.44%, respectively. QTL for all traits but RMST and RNL were identified in the female LGs (Table 2).

**Table 1:** Summarized results of the QTL identified in R43-64 parental map using the data obtained from both growth chamber and field experiments.

QTL name	Linkage group	Traits name	QTL range (cM)	Flanking marker	LOD	Additive value	PVE^a^
**Growth chamber study**							
Qsdrpht.nr-1m	1	RPHT	27–29.20	nffa803-179, nffa803-182	4.00	1.07	18.36
Qsdamst.nr-1m	1	AMST	0.01–10	nffs027-138, nffa448-166	3.10	10.51	14.24
Qsdbmst.nr-2m	2	BMST	24–36	nfmf032-262, nffg009-222	3.07	− 3.02	14.11
Qsdamst.nr-4m	4	AMST	15–33	nffa387-193, nffa689-529	3.00	6.71	13.84
Qsdrmst.nr-4m	4	RMST	27–35	nffa387-193, nffa689-529	2.65	6.24	12.57
Qsdrtn.nr-5m	5	RTN	19–22	nfmf038-163, nffa722-287	3.06	0.98	14.15
Qsdapht.nr-5m	5	APHT	11.40–18.10	nffg110-168, nfmf038-163	4.29	2.18	19.81
Qsdrtn.nr-6m	6	RTN	4–10.50	nffg273-205, nffg170-226	3.50	− 1.11	16.17
Qsdanl.nr-6m	6	ANL	6–10.50	nffg273-205, nffg170-226	3.40	− 1.12	15.69
Qsdbfw.nr-6m	6	BFW	2–11	nffg273-205, nffg170-226	2.45	− 0.38	11.12
Qsdrfw.nr-6m	6	RFW	30–36	nffg264-211, nffa709-464	4.20	− 0.12	19.43
Qsdrdw.nr-6m	6	RDW	30–51	nffg264-211, nfmf100-135	4.01	− 0.06	18.52
Qsdadw.nr-7m	7	ADW	92.90–95.10	nffa610-194, nffa610-194	3.83	0.08	17.77
Qsdrfw.nr-11m	11	RFW	114–130	nffg260-212, nffg222-358	2.25	0.14	10.29
Qsdratw.nr-11m	11	RATW	56–69	nffa826-164, nffa321-109	2.64	− 0.03	12.15
Qsdrnl.nr-12m	12	RNL	123–141.50	nffa069-281, nffa475-103	2.68	− 1.23	11.57
Qsdadw.nr-12m	12	ADW	118–119.50	nffg673-596, nfmf087-159	3.04	− 0.05	14.10
Qsdbmst.nr-12m	12	BMST	12–22	nffg084-181, D561700	3.75	− 3.41	17.25
Qsdbdw.nr-13m	13	BDW	24–55	nffa059-153, nffg366	4.79	− 0.20	22.32
Qsdratw.nr-13m	13	RATW	55–71	nffg366-192, nffg167-108	3.50	− 0.03	16.09
Qsdbmst.nr-17m	17	BMST	6–14	D555144, D561133	3.06	4.56	14.05
Qsdrtn.nr-19m	19	RTN	62–73	nffa635-439, nffa635-412	2.93	− 1.03	13.43
Qsdatn.nr-22m	22	ATN	10–14	nffg301-190, nffg301-172	2.18	1.39	10.03
Qsdanl.nr-22m	22	ANL	15–19.50	nffa050-213, nffa147-221	3.75	1.99	17.14
Qsdrpht.nr-22m	22	RPHT	8–18	nffg301-190, nffa291-527	2.48	− 2.43	11.46
Qsdafw.nr-22m	22	AFW	2–8	nffg301-188, nffg301-190	2.90	− 2.08	13.33
Qsdaatw.nr-22m	22	AATW	0.02–8	nffg301-188, nffg301-190	2.81	− 0.38	13.02
**Field study**							
Qpffw.nr-1m	1	FFW	14–22	nffa448-166, nffa448-170	2.30	78.00	10.58
Qpffw.nr-1m	1	FFW	48–57	nffg273-181, nffg464-168	5.00	96.00	22.86
Qpfpht.nr-1m	1	FPHT	50.20–65	nffg157-111, nffa649-203	3.09	1.36	14.26
Qrsmst.nr-1m	1	SMST	58–69.70	nffg464-168, nffg253-190	2.57	1.83	11.87
Qpsdw.nr-2m	2	SDW	63.50–68.50	nffa112-240, D555477	2.42	− 63.9	11.70
Qpsfw.nr-2m	2	SFW	63.50–67.50	nffa112-240, D555477	2.68	− 171.00	12.40
Qpfpht.nr-3m	3	FPHT	71–73	nfmf176-154, nffg156-172	3.70	2.65	17.08
Qrfmst.nr-3m	3	FMST	24–35	nfmf103-159, nffa692-194	3.90	5.83	18.10
Qrsdw.nr-3m	3	SDW	42–56	nffg389-230, nffa233-202	2.40	33.86	10.95
Qpfdw.nr-5m	5	FDW	11.20–14.60	nffg110-168, nffg009-188	3.67	− 27.50	17.16
Qpffw.nr-5m	5	FFW	11.50–15.10	nffg110-168, nffg009-188	3.16	− 76.00	14.51
Qrsdw.nr-6m	6	SDW	50.20–52.60	nffa370-458, nfmf100-147	3.11	39.54	14.20
Qrsfw.nr-6m	6	SFW	42–52.40	nffg229-178, nfmf100-147	3.75	99.00	17.45
Qrspht.nr-6m	6	SPHT	5.70–17	nffg273-205, D560141	2.30	5.33	10.49
Qrspq.nr-6m	6	SPQ	19.40–44	D561085, nffa370-481	2.60	0.60	11.70
Qpfmst.nr-7m	7	FMST	63.60–69	nffa654-351, D556441	3.50	3.19	15.94
Qpsmst.nr-7m	7	SMST	67.50–69	nffg405-195, nffa205-347	3.90	4.72	17.85
Qpspht.nr-7m	7	SPHT	65.50–71	nffa654-351, D556441	2.37	− 2.37	10.10
Qpspq.nr-7m	7	SPQ	63–71	nffa654-351, D556441	3.34	− 0.77	15.30
Qrsdw.nr-7m	7	SDW	0.50–11	nffg028-150, nffg325-130	2.40	− 31.00	11.05
Qrsdw.nr-7m	7	SDW	35–41	nffg167-109, D555372	2.42	55.00	11.04
Qrsfw.nr-7m	7	SFW	0.40–10	nffg028-150, nffg325-130	2.56	− 88.00	11.75
Qrfmst.nr-8am	8a	FMST	15.50–21.50	nffa206-339, nffa654-379	3.43	8.75	12.14
Qpspht.nr-8bm	8b	SPHT	18–27	nffg026-175, nffg330-225	2.95	− 2.12	13.62
Qpfdw.nr-9m	9	FDW	60–75	nffg236-181, nffg324-186	2.35	− 45.00	10.76
Qpsmst.nr-9m	9	SMST	91.10–95	nffa864-313, nffa864-310	2.77	− 3.78	12.88
Qpspht.nr-9m	9	SPHT	68–76	nffa147-230, nffg324-186	3.57	− 4.20	16.45
Qpfpht.nr-11m	11	FPHT	71–93	nffa826-164, nffa314-170	2.40	− 1.90	11.12
Qrsdw.nr-11m	11	SDW	99–112	nffg101-190, nffa688-125	2.70	− 35.90	12.87
Qrffw.nr-12m	12	FFW	23–35.50	D356378, D557168	2.45	− 54.00	11.17
Qrfpht.nr-12m	12	FPHT	119–133	nffg673-587, nffa069-277	2.52	− 1.24	11.57
Qpsdw.nr-15m	15	SDW	53.4–57	nffa611-154, nffa611-153	5.15	− 72.00	23.74
Qpsfw.nr-15m	15	SFW	49–57	nffa611-154, nffa611-153	2.02	− 112.00	9.35
Qpsmst.nr-15m	15	SMST	12.1–17	nffg258-387, nffa650-502	2.64	4.35	12.10
Qpffw.nr-17m	17	FFW	23–30	nffg328-236, nffg328-235	2.30	76.00	10.64
Qpsfw.nr-17m	17	SFW	23–30	nffg328-236, nffg328-235	2.50	161.00	11.40
Qrsdw.nr-17m	17	SDW	56–60	nffg140-161, D555982*	2.45	− 3.70	11.17
Qrsfw.nr-17m	17	SFW	54–60	nffg140-161, D555982*	2.25	− 79.00	10.22
Qrspq.nr-17m	17	SPQ	54–60	nffg140-161, D555982*	2.45	− 0.53	11.37
Qrsmst.nr-19m	19	SMST	39–43.9	nffa617-117, D561074	2.57	4.36	11.80
Qpfdw.nr-20m	20	FDW	0.50–9	nffg228-160, nffg092-176	2.45	− 33.00	11.14
Qpffw.nr-20m	20	FFW	17–34	D560491, D558624	2.30	− 43.50	10.34
Qpspht.nr-20m	20	SPHT	17.50–22.80	D560491, D558624	3.17	11.80	14.63
Qrffw.nr-20m	20	FFW	32–34	D558624, D555476	3.30	− 178.00	15.04
Qrfpht.nr-20m	20	FPHT	0.5–6	nffg228-160, nffg092-176	3.32	− 2.32	15.24
Qrsmst.nr-20m	20	SMST	20.80–27.70	D560491, D558624	5.02	9.20	22.92
Qrspht.nr-20m	20	SPHT	19.5–28	D560491, D558624	5.09	15.00	23.50
Qrfdw.nr-22m	22	FDW	10.20–11.50	nffg301-190, nffg249-181	2.53	39.17	11.69
Qrffw.nr-22m	22	FFW	10.20–18	nffg301-190, nffa524-325	2.78	108.00	12.87
Qrspq.nr-22m	22	SPQ	14–17.80	nffg301-172, nffa469-286	2.40	− 1.41	11.08

The QTL name starts with a “Q”, followed by phenotyping procedure (“sd” phenotyping for summer dormancy in growth chamber, “p” PDF field location and “r” research park field location), abbreviated trait name (ATN—after cut back tiller number; ANL—after cut back new leaf; APHT—after cut back plant height; AFW—after cut back fresh weight; ADW—after cut back dry weight; AMST—after cut back moisture content; AATW—after cut back average tiller weight; RTN—return to normal growth tiller number; RNL—return to normal growth new leaf; RPHT—return to normal growth plant height; RFW—return to normal growth fresh weight; RDW—return to normal growth dry weight; RMST—return to normal growth moisture content; RATW-return to normal growth average tiller weight; BFW—before cut back fresh weight; BDW—before cut back dry weight; BMST—before cut back moisture content;. SPQ—spring plant quality; SPHT—spring plant height; SFW—spring fresh biomass weight; SMST—spring plant moisture content; SDW—spring dry biomass weight; FPHT—fall plant height; FFW—fall fresh biomass weight; FDW—fall dry biomass weight; FMST—fall plant moisture content.), abbreviated name of the institute (“nr” Noble Research Institute), linkage group number (1 to 22) and ends with parental origin of the LGs (“f” female and “m” male). LOD—logarithm of odds for additive genetic effect.

^a^*PVE* phenotypic variance explained.

**Table 2:** Summarized results of the QTL identified in 103-2 parental map using the data obtained from both growth chamber and field experiments.

QTL name	LG	Traits name	QTL range	Flanking marker	LOD	Additive value	PVE
**Growth chamber study**							
Qsdatn.nr-3f.	3	ATN	29–35	nffg170-224, nffg273-194	2.23	1.04	10.31
Qsdbmst.nr-3f.	3	BMST	42–50	nffg273-194, nffa155-211	2.40	− 3.28	11.01
Qsdanl.nr-4af	4a	ANL	50–58	nffa788-298, nffg335-177	2.20	1.10	10.05
Qsdrdw.nr-4af	4a	RDW	15–25	D558615, D355986	2.35	0.05	10.76
Qsdanl.nr-4bf	4b	ANL	12–30	nffg391-147, nffa295-239	2.18	− 0.89	9.91
Qsdrtn.nr-5f.	5	RTN	19–40	D560563, D555824	3.11	− 1.56	14.22
Qsdbfw.nr-5f.	5	BFW	4–30	nffa877-277, D557383	2.35	− 0.37	10.80
Qsdadw.nr-6af	6a	ADW	0.01–21	D562378, nffg171-143	3.86	− 0.08	17.83
Qsdrpht.nr-7f.	7	RPHT	65–85	nffa773-126, nffa206-321	3.07	1.09	14.34
Qsdbdw.nr-7f.	7	BDW	90–94	nffa206-321, nffg106-176	2.50	0.19	11.51
Qsdapht.nr-12af	12a	APHT	2–14	nffg332-230, D556156	2.40	2.20	10.82
Qsdadw.nr-12af	12a	ADW	6–23	nffg332-230, D562496	3.45	0.08	15.88
Qsdatn.nr-13f.	13	ATN	67–80	nfmf088-168, D561075	2.20	− 1.18	10.06
Qsdrtn.nr-13f.	13	RTN	69–80	nfmf088-168, D561075	3.50	− 1.27	16.28
Qsdratw.nr-14bf	14b	RATW	0.10–5	nffa787-580, nffa635-431	2.30	− 0.03	10.31
Qsdatn.nr-14cf	14c	ATN	7–16	nffg295-173, nffa800-181	3.65	− 1.62	16.84
Qsdrtn.nr-14cf	14c	RTN	11–18	nfmf050-181, nfmf228-287	3.47	− 1.61	16.10
Qsdrfw.nr-14cf	14c	RFW	13–16	nffa635-388, nffa800-181	3.52	0.28	16.20
Qsdratw.nr-14cf	14c	RATW	13–16	nffa635-388, nffa800-181	2.40	0.04	10.98
Qsdrpht.nr-20f.	20	RPHT	18–46	nffa802-496, D561092	2.45	1.28	11.20
Qsdamst.nr-22f.	22	AMST	0.5–10	nffa677-316, nffs205-564	2.68	− 8.32	12.33
Qsdafw.nr-23f.	23	AFW	16.10–16.70	nffa635-433, nffa671-230	4.67	− 4.10	21.44
Qsdrfw.nr-23f.	23	RFW	2–14	nffa360-464, nffa709-441	2.70	− 0.19	12.45
Qsdaatw.nr-23f.	23	AATW	16.1–16.7	nffa635-433, nffa671-230	4.60	− 0.78	21.27
**Field study**							
Qrfdw.nr-5f.	5	FDW	19–40	D560563, D562270	2.20	− 31.0	10.10
Qpspht.nr-13f.	13	SPHT	0.3–8.5	nffa870-167, nffa864-335	4.03	3.28	17.44
Qrfpht.nr-13f.	13	FPHT	47–53	nffa864-337, nffa346-335	2.35	1.67	10.67
Qrspq.nr-15f.	15	SPQ	31–35	D559702, D356277	3.82	0.80	17.62
Qpfdw.nr-17f.	17	FDW	74–80	D562318, nffa694-119	2.88	− 65.0	13.30
Qpffw.nr-17f.	17	FFW	74.90–82.20	D562318, nffa694-119	3.54	− 247	16.30
Qrfdw.nr-17f.	17	FDW	78–84	D562318, nffa694-119	3.72	− 64.0	17.28
Qrffw.nr-17f.	17	FFW	80–84	D562318, nffa694-119	6.00	− 340	27.60
Qrsmst.nr-17f.	17	SMST	79–83	D562318, nffa694-119	7.05	8.80	32.67
Qrspq.nr-17f.	17	SPQ	16–30	ctg023-483, nfmf192-198	2.78	0.63	12.88
Qrfmst.nr-20f.	20	FMST	67.30–87	nffa147-218, nffa699-639	2.55	− 4.56	11.71
Qrfpht.nr-22f.	22	FPHT	10.10–20	nffs205-564, nffg325-111	2.20	− 1.30	10.00
Qrspq.nr-22f.	22	SPQ	17–37	nffs205-564, nffg325-111	2.92	− 0.92	13.56
Qpspq.nr-14bf	14b	SPQ	7.3–10.3	nffg106-174, D561372	3.75	1.75	17.45
Qrsmst.nr-14bf	14b	SMST	8.40–11	nffg106-174, D561372	2.89	3.36	13.28
Qpfdw.nr-14cf	14c	FDW	12.50–15.60	nffa460-140, nfmf016-199	3.81	43.0	17.57
Qpfpht.nr-14cf	14c	FPHT	13.50–15.50	nffg253-228, nfmf016-199	4.50	4.91	20.68
Qrsdw.nr-14cf	14c	SDW	15–19.40	nffa314-141, nffg241-173	2.48	128	11.34
Qrsfw.nr-14cf	14c	SFW	15–19.30	nffa314-141, nffg241-173	3.17	125	14.63
Qrsmst.nr-19af	19a	SMST	0.50–8.30	D560458, D555552	4.30	8.44	19.65
Qrfdw.nr-19bf	19b	FDW	40–45	nffa772-453, nffa634-346	2.90	− 46.49	13.42
Qrsmst.nr-4af	4a	SMST	31–37	nffg197-166, D555867	2.30	− 2.52	10.60

The QTL name starts with a “Q”, followed by phenotyping procedure (“sd” phenotyping for summer dormancy in growth chamber, “p” PDF field location and “r” research park field location), abbreviated trait name (Abbreviation of the traits names are same as provided in Table 1), abbreviated name of the institute (“nr” Noble Research Institute), linkage group number (1 to 23) and ends with parental origin of the LGs (“f” female and “m” male).

QTL associated with important traits are summarized in Tables 1 and 2. BFW QTL was colocalized with a RTN and an ANL QTL on LG 6 of the R43-64 map. All the three QTL had negative additive effect and flanked by nffg273-205 and nffg170-226 SSR markers. The correlation analysis of these three traits showed significant positive correlation among the traits meaning that the QTL might have pleiotropic effect for RTN, ANL and BFW. Additive effect of the QTL also interprets that these QTL contribute to increase TN, NL and FW of the plants growing under summer dormant condition. QTL detection in growth chamber study was done using the data obtained from the difference between optimum and summer dormant conditions. Thus, lower value indicates less phenotypic response variability between the two conditions.

It was evident that non-dormant plants tend to continue growing under dormancy condition unless a severe stress effect is imposed^1^. QTL region located in the Continental map made it meaningful that this region might have been inherited from the parent and contribute for stress tolerance without enhancing summer dormancy. Another fresh weight (RFW) QTL was colocalized with a RDW QTL between 30 and 36 cM regions of LG 6. Both the QTL had negative additive effect. RFW and RDW had significantly positive but low correlation (0.48) than usual. The low correlation between FW and DW under the same environmental condition might be due to variability of moisture content in plants, which was the result of either summer dormancy or stress response. The colocalization of BDW and RATW QTL on LG 13 also signify the importance of the QTL as both traits were measured in two different regrowth periods. In both the cases, high PVE support the strength of the QTL. Negative additive effect shows that this QTL influenced the plants to increase BDW and RATW under summer dormant and return to normal growth conditions, respectively, which is a phenomenon of Continental tall fescue under moderate stress with ample water availability^1^.

In the female parental LGs, QTL having positive additive effect under summer dormancy condition and colocalized with QTL under return to normal growing condition were not detected. However, QTL associated with APHT (Qsdapht.nr-12af) and ADW (Qsdadw.nr-12af) in LG 12 showed positive additive effect, colocalized and appeared under summer dormant condition. Positive additive effect of the QTL explains plant height and DW reduction under summer dormant condition, which might be due to either of summer dormancy, stress or both together. QTL region on LG 14c was colocalized with QTL associated with ATN, RTN, RFW and RATW. The additive effect of the QTL indicate that the region tends to hold TN as usual under both summer dormant and optimum growing conditions. Meanwhile the same region also affects the plants to reduce FW under return to normal growth condition after passing the dormant condition. This become meaningful that this region influences the plants not to be dormant and thus there was a carryover stress effect in FW reduction even under normal growing condition after the dormancy stress.

### QTL detection in the field experiment

A total of 50 QTL were identified in male parental LGs with LOD and PVE value ranged between 2.02–5.15 and 9.35–23.74, respectively (Table 1). Highest number of QTL (7) was found in LG 7 and LG 20 followed by LG 17 (5). The highest number of colocalized QTL (5) was found in LG 20 followed by LG 7 (4). Considering field and growth chamber study, common QTL regions were found in; 11.2–18.1 cM region of LG 5 and 30.0–53.6 cM of LG 6; 118.0–141.5 cM of LG 12 and 8.0–19.5 cM of LG 22 (Table 1). Female LGs were found to be associated with 22 QTL. The LOD values of the QTL ranged from 2.2–7.05 with the PVE ranged from 10.10 to 32.67 (Table 2). QTL Qrsmst.nr-17f. explained the highest PVE (32.67) followed by Qpfpht.nr-14cf (20.68) with the LOD threshold of 7.05 and 4.5, respectively. The largest number of colocalized QTL (5) was found in LG 17 followed by LG 14C (4) in the region of 74.0–84.0 cM and 12.5–19.4 cM, respectively. The common QTL regions found between the growth chamber and field study were located in 19.0–40.0 cM region of LG 5 and 7.0–19.4 cM region of LG 14C (Table 2).

All the QTL in LG 6 showed positive additive effect for spring FW, DW, PHT and PQ. This indicates that the QTL has positive influence on the traits (as direct mean data were used in QTL analysis for field traits). Increment of growth related traits during spring is a typical growth pattern of Continental tall fescue^19^. In both field studies the QTL were detected when summer dormancy period was over and contributed positive attributes to the plants. Thus, the QTL might have pleotropic effect for summer dormancy, stress adaptation and biomass enhancement under optimum growing condition. Even though the QTL was not declared as summer dormant QTL, this is a potential QTL region to be used for MAS in tall fescue breeding.

There were four colocalized QTL in LG 7 of male parental map (Table 1); two of which showed positive additive effect for PDF spring moisture content (PSMST) and PDF fall moisture content (PFMST) and another two showed negative additive effect for PDF spring plant height (PSPHT) and PDF spring plant quality (PSPQ) meaning that this QTL region tends to increase plant moisture under both summer and spring conditions and reduced PHT and quality under spring condition. Practically, summer dormant plant should show opposite response by decreasing growth performance during summer and increasing performance after summer. However, no QTL was identified in this region in the growth chamber study. Based on the hypothesis, the QTL may not be identified as summer dormancy related QTL, but it might be a strong stress responsive QTL.

Female parental LG 14C was found associated with four colocalized QTL (Table 2) for FDW, FPHT, SDW and SFW. Positive additive effect of all the QTL explains growth enhancing influence of the QTL region under both summer and spring conditions. Based on the growth chamber study, the attributes were related to the Continental tall fescue. The flanking marker of the QTL region might be used for enhancing yield gain of the Continental tall fescue.

### Putative summer dormancy QTL

Summer dormancy is a conjugated response of long day, high temperature and drought; thus, it is highly difficult to differentiate QTL associated with summer dormancy from the other stress related response in the uncontrolled field study. Thus far, QTL identified using both growth chamber and field studies were aligned and compared to identify the QTL associated with summer dormancy. Before and after cut back condition in growth chamber study were comparable to summer condition in field study and return to normal growth condition of growth chamber study was comparable to spring time in field study.

**Figure 3:**
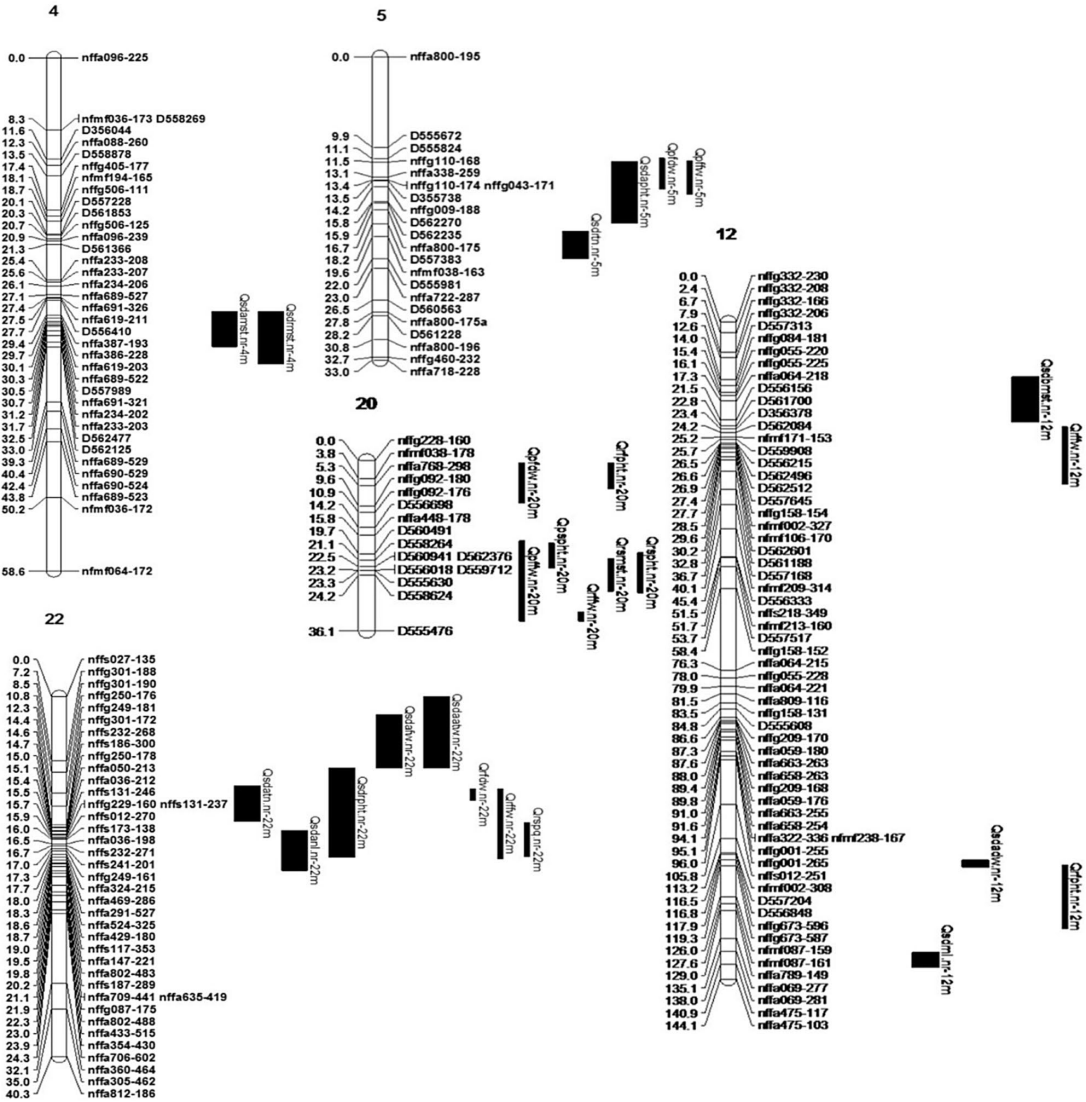
Putative summer dormancy related QTL identified in male parent (R43-64) map. Narrower bars indicate QTL identified in field study and wider bars indicate QTL identified in growth chamber study. QTL name starts with a “Q”, followed by phenotyping procedure (“sd” summer dormancy in growth chamber, “p” PDF farm and “r” Research Park field), abbreviated trait name (e.g., BMST-before cut back moisture content), institute name (“nr” Noble Research Institute), LG number (1 to 22) and ends with parental origin (“f” female and “m” male parent map).

**Figure 4:**
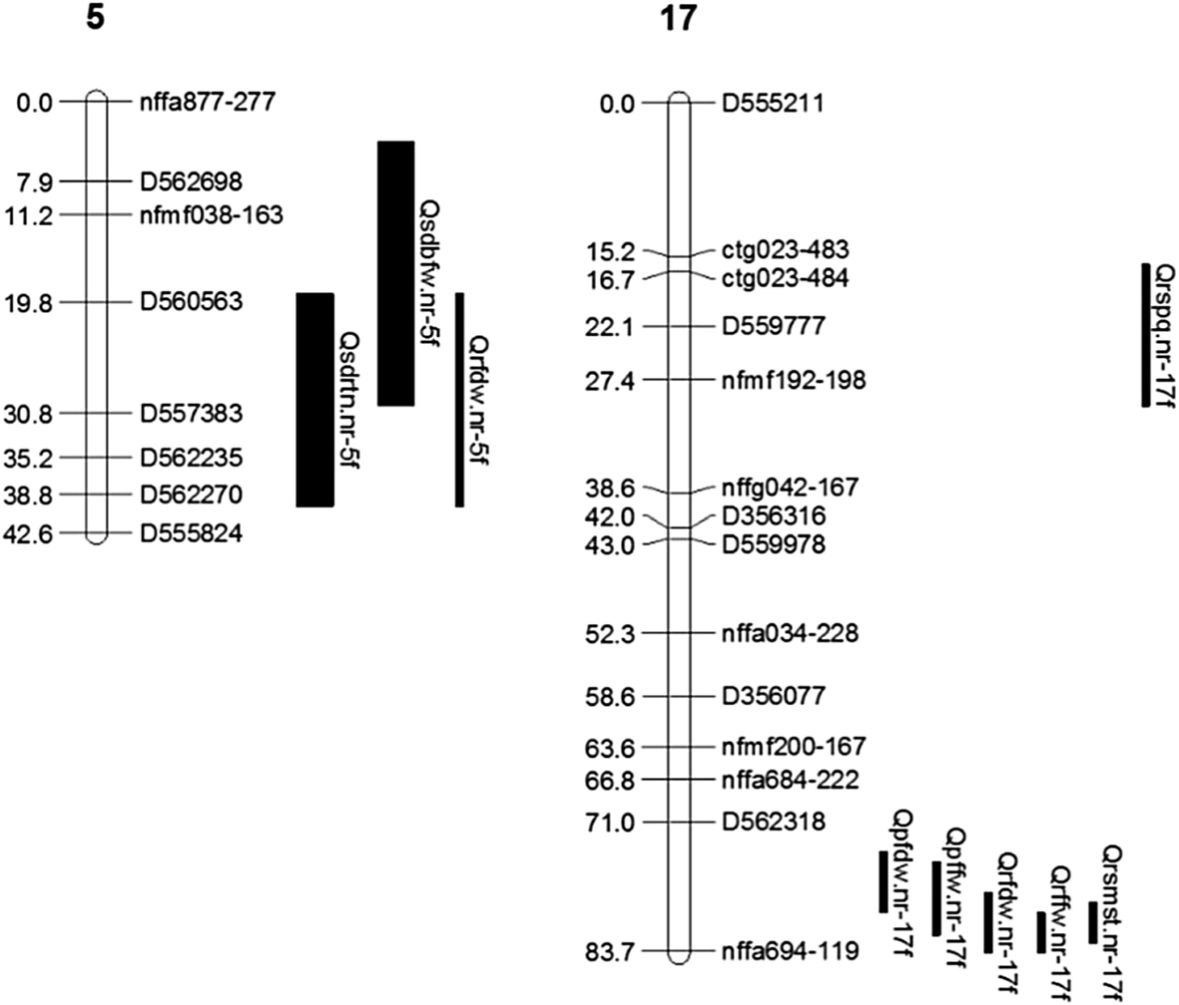
Putative summer dormancy related QTL identified in female (103-2) map. Narrower and wider bars indicate QTL identified in field and growth chamber studies, respectively. QTL name starts with “Q”, followed by phenotyping procedure (“sd” summer dormancy in growth chamber, “p” PDF farm and “r” research park field), abbreviated trait name (e.g., bfw-before cut back fresh weight), abbreviated institute name (“nr” Noble Research Institute), linkage group number (1 to 23) and ends with parental origin of the LGs (“f” female and “m” male parent map).

Due to the confounding effect of dormancy and stress to the plants, we had set the hypothesis that QTL region appeared to be associated with reducing plant performance only under dormancy condition might be either of stress or dormancy responsive QTL. To distinguish a summer dormant QTL from a stress responsive one, the hypothesis was that summer dormant QTL should show pleiotropic response for growth reduction and enhancement during summer dormant and return to normal growing conditions, respectively. In a long term dormancy, plants might be highly responsive to summer stresses^37^ and continued to be dormant for a while even after the dormancy condition is over. We considered that if a dormancy QTL showed positive additive effect under return to normal growth condition in the growth chamber study might be associated with long-term dormancy and showing negative additive effect might be associated with short-term dormancy. Thus, we considered four assumptions as discussed in materials and methods to declare summer dormant QTL. Based on the assumptions, QTL in LGs 4, 5, 12, 20 and 22 of male parental groups (Fig. 3) and LGs 5 and 17 of female parental groups (Fig. 4) were found associated with summer dormancy. The rest of the QTL has potential to be related to stress response, summer dormancy or normal agronomic traits.

Phenotyping for summer dormancy is critical and no QTL study has been done yet for summer dormancy in tall fescue. As a result, we had limited ability to verify the reported QTL based on previous findings. It was defined earlier that summer dormancy should be measured by quantifying senescence^1^. In the growth chamber study, there was no complete senescence due to watering for plant survival and it was not possible to quantify senescence level among the genotypes. Thus, moisture content was treated as the secondary trait of senescence. To measure complex trait like summer dormancy or senescence, secondary traits might be highly useful. Secondary traits were found effective in improving drought tolerance in maize^38^. In a field study with *Miscanthus*, senescence was found strongly correlated with biomass moisture content during harvest^39^. AMST and RMST were moisture content traits at two different regrowth periods under summer dormant and normal growth conditions, respectively. Positive additive effect of the AMST QTL in LG 4 denoted that this QTL have contributed to reduce biomass-moisture-content under summer dormant condition and thus enhanced senescence, as well as summer dormancy. Similarly, RMST-QTL showed the same effect under return to normal growth condition. In a study using orchard grass, bulbous barley and hardinggrass^40^, it was observed that those grasses remained dormant for 4 months even with full irrigation during summer, which they defined as physiological dormancy. Thus, the QTL region associated with AMST and RMST in this study may enhance physiological dormancy in tall fescue.

The QTL region in LG 5 was appeared in both growth chamber and field study (Fig. 3). In the growth chamber study, the QTL tended to reduce plant height and tiller number under dormancy and return to normal growth condition, respectively. The same QTL region in the field showed reducing response of fresh and dry weight during fall harvest, which reflected the negative growth response of plants during summer. The response of growth reduction under both dormancy and return to normal condition might be associated with long term physiological summer dormancy^37^. Tall fescue exhibit only partial summer dormancy^1^, thus observing mixed dormancy and stress effect is highly likely. Carryover stress effect also can be reflected during return to normal growth condition.

Negative additive effect of fall fresh weight QTL (Qrffw.nr-12m) in male LG 12 might be due to stress response, as the same QTL in the growth chamber study tends to increase the moisture level under dormancy condition. Meanwhile, the FPHT QTL (Qrfpht.nr-12m) might be interesting. The negative additive effect showed reduction of plant growth in summer, while the same QTL region showed increased RNL during return to normal growing condition after experiencing a dormancy spell in growth chamber study. All these results suggest this may be an interesting region in tall fescue genome controlling the summer dormancy. QTL region (17–34 cM) in male LG 20 was colocalized with five QTL and had reduced fresh biomass weight during summer. The same QTL region showed positive influence on enhancing plant moisture content and plant height during spring meaning a pleotropic response of the region between summer and spring (Table 1). Based on pleiotropic response criteria^1,6,28^, this might be a potential QTL contributing to summer dormancy. LG 22 was found associated with five QTL in growth chamber study associated with reducing tiller number and new leaf under dormancy condition while increasing plant height at return to normal growth condition. The QTL might be associated with summer dormancy. Positive additive effect of fall fresh and dry weight might be the result of short-term dormancy and pleiotropic genetic control of the region. Negative additive effect of spring plant quality of the same QTL region at field study, also denotes that this QTL might be associated with summer dormancy. Because summer dormant plants may show poor survival during cold spring^41^.

QTL in LG 5 of female parental map seems interesting. Negative additive effect of the QTL in field study indicates the tendency of decreasing dry biomass weight due to summer, while the same region showed tendency of increasing tiller number at return to normal growth condition in growth chamber study (Table 2). This phenomenon is likely to be associated with summer dormancy. QTL region in LG 17 identified in the field study was found to be associated with summer dormancy. The negative additive effect of fall fresh and dry weight in both locations indicated that, this QTL is associated with reducing fresh and dry weight during summer (Table 2). The same QTL region had positive additive effect for SMST and spring plant quality meaning that the QTL is associated with increasing plant performance when the dormancy effect was over. Comparing with the QTL effect in LG 22 of male map, this QTL region might be associated with long term or physiological dormancy thus dormancy effect continued till the fall harvest. According to Varshney et al.^42^ and Zhao et al.^43^, QTL can be considered stable if they appeared in more than one location. Thus, the QTL might be an important and stable one for long-term summer dormancy, as it was consistently detected across locations with high LOD and PVE values.

Expression of summer dormancy in tall fescue is not as prominent as orchard grass^1,40^; thus dismantling dormancy mechanism from the stress response would be very critical due to the confounding effect of both the phenomena. We found significantly lower amount of marker distribution, in the female parental maps, which might result for yielding fewer QTL associated with summer dormancy. However, higher marker coverage and transgressive segregation of the phenotypic traits supports the identification of reasonable amount of summer dormancy QTL in the summer active parental map. Indeed, saturating the population with high-density marker will enhance QTL mapping and marker detection for summer dormancy to use in MAS.

### Epistasis among the QTL

**Figure 5:**
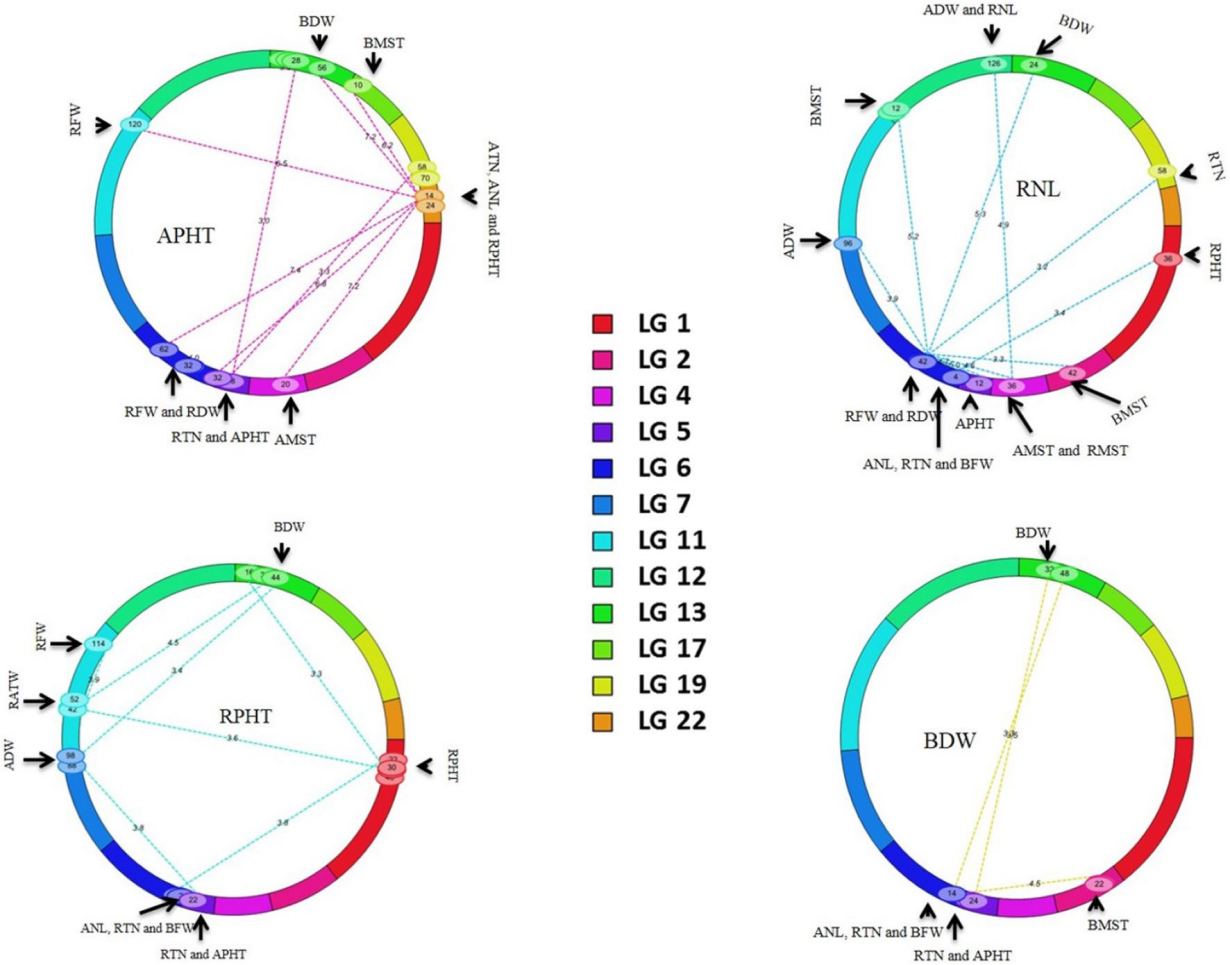
Epistatic interaction among various QTL regions in male (R43-64) parental LGs based on growth chamber study. Each circle indicates epistatic interaction for single trait which is written in the middle of the circle. Different color of the circle represents the interacting LGs. The LG names with color legend are provided in the middle. The connecting line shows the interaction between the LGs. The number on the LGs denotes the centiMorgan (cM) position of interacting regions. The number on the connection line represents LOD threshold of significance. The traits name outside the circle are the previously detected QTL position associated with those traits.

Significant epistasis was found associated with APHT, RNL, RPHT and BDW in the R43-64 parental maps (Fig. 5), and AFW, ADW, AMST, AATW and RTN in the 103-2 maps (Supplementary Fig. S4). Compared to the 103-2 LGs, male LGs showed higher number of epistatic interactions might be due to lack of marker coverage in the female LGs, which is completely in agreement with QTL detection phenomena encountered in this study. Till now, most epistatic studies have been involved with plant height, ear height, total leaf number per plant, ear leaf length, ear leaf width, ear leaf area, and other agronomic traits^44^. Summer dormancy is a very complex phenomenon exhibited by the plants under multiple abiotic stress conditions. The concurrent exposure of a plant under various stress condition would result in the co-activation of various stress response pathways having synergistic or antagonistic effect on each other^45^. In this study, we tried to exploit plant height, tiller number, leaf number, fresh weight, dry weight and moisture content of the plants as phenotypic response to summer dormancy. Phenotypic correlation analysis of the traits showed significant correlations between/among the traits and thus significant epistasis among the QTL region of the LGs not only proves that identified QTL regions cross talks as dormancy/stress response, but also amplifies the reliability of the QTL detected in the regions. As example, Qsdrfw.nr-6m and Qsdrdw.nr-6m QTL in LG 6 could not be explained as putative summer dormant QTL. However, the regions showed interaction with putative summer dormant QTL in LG 4, 5 and 12 along with other stress related QTL for RNL trait. Similarly, QTL region in male LG 22 showed significant epistasis for plant height and interacting with seven other QTL regions including two other putative QTL regions for summer dormancy meaning that, there are pleiotropic effects of the QTL for stress response and summer dormancy. Some of the stress responsive QTL might trigger summer dormancy as an adaptive phenomenon under certain environmental changes by cross talking each other. Thus, the phenomenon of exhibiting summer dormancy in tall fescue requires not only long days but also high drought stress to activate dormancy related genes through cross talk with stress responsive genes.

## Conclusion

We have improved the previously reported linkage maps of the 103-2 × R43-64 tall fescue population with additional SSR marker. The maps have been used to identify QTL associated with summer dormancy related traits. Putative QTL have been identified for fresh weight, dry weight, plant height, tiller number and plant moisture content. Because summer dormancy and summer stress in tall fescue is very difficult to dissect, we hypothesized four assumptions to define QTL’s association with summer dormancy. Based on those assumptions, seven QTL were identified which might have potential contribution to summer dormancy in tall fescue. This is the first bi-parental QTL mapping research article in tall fescue summer dormancy and second for summer dormancy QTL mapping in any crop. Phenotyping summer dormancy in tall fescue is highly difficult due to effects of exogenous conditions. Thus, the molecular marker associated with summer dormancy related QTL identified in this study, will be valuable resources to the tall fescue breeders to initiate marker-assisted breeding, which will facilitate improved cultivar development with adequate summer dormancy.

## Materials and methods

### Plant materials

A pseudo F_1_ testcross population was developed by crossing between a Mediterranean parent, 103-2, and a Continental parent, R43-64, as described by Dierking et al.^24^. Under extreme summer conditions, the 103-2 exhibits complete summer dormancy, while the R43-64 experiences stand loss due to continuous growth habit. Two-hundred-two F_1_ progenies obtained from the cross were used for trait evaluations, of which 195 were used for genotyping using SSRs and 108 with DArT marker. All plants derived from this cross were maintained in the Noble Research Institute, Ardmore, OK, USA greenhouse as clonal ramets.

### Plant care and experiment under growth chamber condition

**Figure 6:**
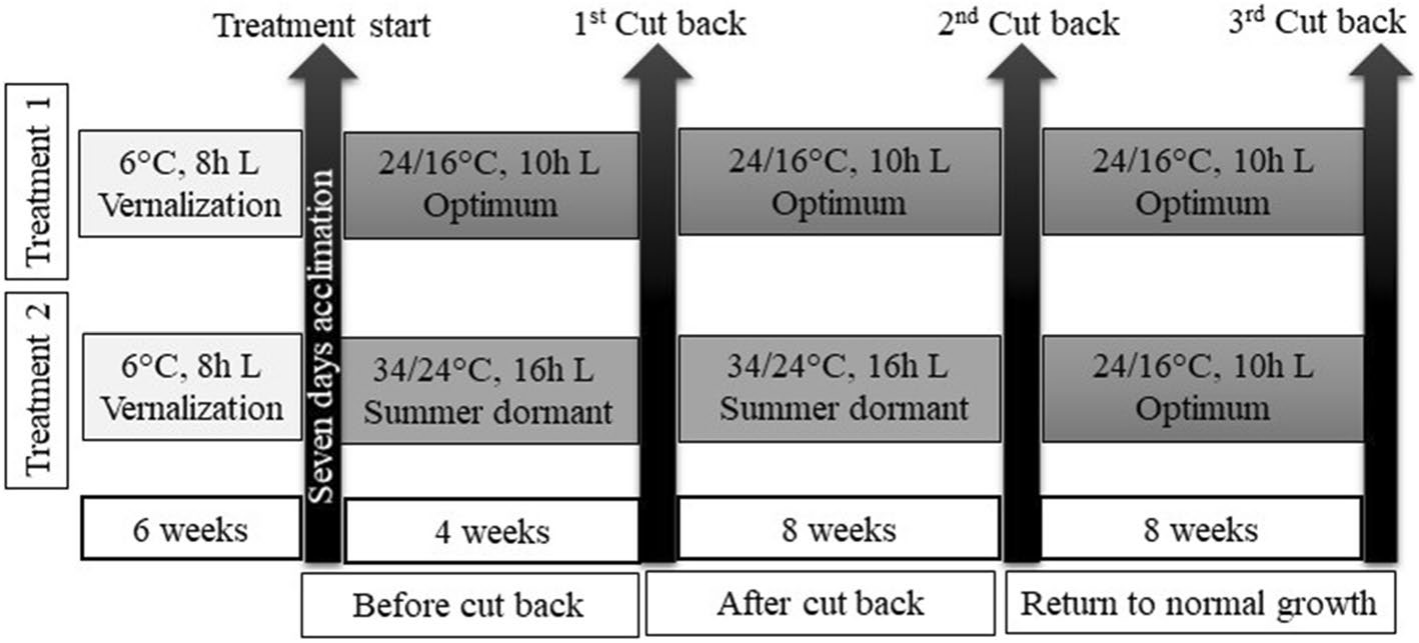
Schematic diagram of the growth chamber study indicating growth conditions and time of data collection. In both the treatments, plants were kept for 7 days at 21/16 °C day/night temperature and 10 h photoperiod for acclimatization after vernalization. In treatment 1, plants were continuously kept under optimum growing condition for all three-cutbacks. In treatment 2, plants were kept under summer dormant condition for 1st and 2nd cut back and under optimum growing condition for 3rd cut back. Data were collected during three different cutbacks.

Clonal ramets of the parents and 202 progenies were planted in 30 cm cones maintaining two tillers per cone and allowed to establish in the greenhouse for 2 weeks. After establishment, the plants were moved into vernalization chamber and vernalized for 6 weeks at 6 °C and 8 h photoperiod. Plants were then taken into growth chambers and acclimatized for 7 days at 21/16 °C day/night temperature and 10 h photoperiod. There were two treatments in the experiment and three growing phases under each treatment. The first growing phase continued for 4 weeks and was termed as “before cut back”. The second phase continued for 8 weeks and was termed as “after cut back”. The third phase continued for 8 weeks and was termed as “return to normal growth”. In treatment one, all three growing phases were maintained for optimum growth. In treatment two, “before cut back” and “after cut back” phases were maintained at summer dormant condition and “return to normal growth” phase was maintained for optimum growth (Fig. 6). In both treatments, watering was done carefully, so that plants did not suffer for water stress. Total of four growth chambers were used assigning two chambers for each treatment. The experiment was conducted following split plot arrangement in Randomized Complete Block design (RCBD) with two replications/blocks where treatments were used as main plot and genotype as subplot.

At the end of every phase, plants were given a cutback. Thus, three cutbacks were done and data were taken before each cut, which reflected the response of relevant phase of the cutback. The 1st cut was relevant to “before cut back” phase and data were taken on BFW, BDW and BMST. The 2nd cut was relevant to “after cut back” phase and data were taken on ATN, ANL, APHT, AFW, ADW, AATW and AMST. The 3rd cut was relevant to “return to normal growth” and data were taken on RTN, RNL, RPHT, RFW, RDW, RATW and RMST.

### Field planting, experimental design and trait measurement

The population was also phenotyped in two field locations; the Research Park (latitude 34.11° N, longitude 97.54° W) and Pasture Demonstration Farm (PDF, latitude 34.13° N, longitude 97.13° W), at Ardmore, OK. Clonal ramets of 202 full sib progenies along with two parents were planted in finely prepared seedbeds following RCBD with three replications at both the locations. The clonal ramets were considered as the experimental unit. Both the experimental locations were planted during mid-October 2008 maintaining plant and row spacing to one meter. Supplemental irrigation and NPK fertilization were done following soil test index recommendation of Oklahoma State University (www.osufacts.okstate.edu). Harvesting was done twice a year; during the first week of May as spring harvest and during the first week of October as fall harvest. Data taken in 2010 spring and fall growth period were used for QTL mapping. Plant quality (PQ) data were taken only during spring at both locations by scoring them in a 0 to 10 scale. The best plant based on vigor and stay-green was given “10”, while the senesced or seemingly dead one was given “0”. PHT was measured from base to the top most leaf/panicle of each plant in centimeters before spring and fall harvest, respectively. FW was taken during the harvests. DW was taken after 72 h of oven-drying at 45 °C. Plant moisture content (MST) was calculated using the following formula:

$$ {\text{Moisture}}\;{\text{content}} = \left( {{\text{Fresh}}\;{\text{weight}} - {\text{dry}}\;{\text{weight}}} \right)/{\text{fresh}}\;{\text{weight}}*{1}00. $$

All the field traits names are abbreviated; preceded by first letter of the location and/or season name, i.e. Research park spring plant quality—RSPQ, PDF spring plant quality—PSPQ, Research park spring plant height—RSPHT, Research park fall plant height—RFPHT, PDF spring plant height—PSPHT, PDF fall plant height—PFPHT, Research park spring fresh weight—RSFW, Research park fall fresh weight—RFFW, PDF spring fresh weight—PSFW, PDF fall fresh weight—PFFW, Research park spring dry weight—RSDW, Research park fall dry weight—RFDW, PDF spring dry weight—PSDW, and PDF fall dry weight—PFDW.

### Statistical analysis

Phenotypic data were analyzed using SAS 9.3 (2011) statistical program (SAS Institute, Cary, NC). Data normality was checked using PROC UNIVARIATE procedure of SAS 9.3. A generalized linear model (GLM) analysis of variance (ANOVA) was conducted for growth chamber data. Phenotypic correlation and simple regression was calculated for the growth chamber data using PROC corr procedure of SAS 9.3. A mixed model ANOVA was conducted for field experiment data considering location as fixed effect and replication within location, genotype and genotype*location as random effects. Statistical significance was determined at *P* ≤ 0.05.

### DNA extraction, marker profiling and linkage map construction

Young leaf tissue from the 202 pseudo F_1_ progenies along with two parents were collected in a 2-ml tube and immediately frozen in liquid nitrogen. Samples were ground to fine powder using a Mixer Mill Type MM 300 (Retsch, Hann, Germany). DNA extraction, quantification and quality check procedures were described in Dierking et al.^24^. To simplify the genotyping complicacy, each polymorphic fragment was considered as independent marker. Total of 1968 molecular markers were used for linkage mapping, among which 1,381 and 587 were SSR and DArT markers, respectively. All the DArT and 924 SSR marker information was taken from Dierking et al.^24^. The final marker groups included tall fescue EST-SSRs (NFFA) and genomic SSRs (NFFG), conserved grass EST-SSRs (CNL), meadow fescue EST-SSRs (NFMF) and STS-CTG (NFFS). Linkage maps were constructed independently using both dominant and heterozygous marker for each parent using JOINMAP 4.0^46^ (https://www.kyazma.nl/index.php/JoinMap/) following cross pollinator (CP) mapping strategy. Both the distorted and non-distorted marker were used for map construction, if addition of distorted markers did not severely change the group positions. We observed 32 pairs of closely linked or cosegregated markers in the data set. Half of these markers were from different marker source. Inclusion of these markers did not affect marker position or map length, thus all the markers were kept during map construction. LGs were selected with a LOD grouping threshold of 5.0 and above. The details of the marker description, genotyping procedure and mapping strategies were similar to Dierking et al.^24^.

### QTL analysis

QTL analysis was conducted using the windows version of QTL Cartographer V2.5^47^ (https://brcwebportal.cos.ncsu.edu/qtlcart/WQTLCart.htm). Difference of the mean data between treatments one and two in growth chamber study (Supplementary Table S3) were used for QTL analysis. For the field study, both season and location mean data were independently used for QTL analysis. Initial approach was to see any marker trait association using single marker analysis. QTL were then identified and reported using the composite interval mapping (CIM). The CIM was performed based on model 6 using forward and backward regression method as a cofactor to control genetic background during position testing in the genome at a threshold of *p* < 0.05. Cofactor selection was done using the window size of 10 and walking speed of 2.0 along the LG. The genome threshold of significance (*p* < 0.05) of the QTL were calculated by 1,000 times permutation analysis and was ranging between 3.09 and 3.71 for all the traits. We considered single QTL for reporting with LOD score of 2.5 or above. QTL fulfilling the declaration criteria and colocalized with other traits were also reported with minimum LOD of 2.0^48,49^. QTL naming follows the Rules of Genetic Nomenclature (https://wheat.pw.usda.gov/ggpages/wgc/98/Intro.htm). The linkage groups having significant QTL in growth chamber study were considered for epistasis analysis using QTL IciMapping V4.0^50,51^ (https://www.isbreeding.net/software/?type=detail&id=15) by selecting the ICIM-EPI model with a probability value for entering variables (PIN) of 0.0001. Significant epistasis between/among the QTL regions was reported based on the default threshold LOD of 3.0 in ICIM-EPI QTL detection.

QTL fulfilling any one of the following four criteria were considered as putative summer-dormancy related QTL: (1) QTL detected in summer dormant condition having positive additive effect and colocalized with QTL under return to normal growth condition with either positive or negative additive effect in the growth chamber study, (2) QTL identified using any fall trait having negative additive effect and colocalized with any spring trait with positive additive effect in the field study, (3) QTL having positive additive effect under summer dormant condition in growth chamber study colocalized with positive additive effect of spring traits QTL in field study and (4) QTL having negative additive effect of fall traits in field study colocalized with QTL showing negative additive effect under return to normal growth condition in growth chamber study.

## Supplementary information

Supplementary Information.

## Data Availability

The datasets used and/or analyzed can be obtained from the corresponding author on reasonable request.
