# Supplementary material for: Mapping QTL for summer dormancy related traits in tall fescue (Festuca arundinacea Schreb.)
Source: Sci Rep. 2020 Sep 3;10:14539. doi: 10.1038/s41598-020-71488-8 (PMC7471293; doi:10.1038/s41598-020-71488-8)
Supplement: Supplementary file 1 — Supplementary Information. [file 41598_2020_71488_MOESM1_ESM.pdf]

Mapping QTL for summer dormancy related traits in tall fescue (*Festuca arundinacea* Schreb.)

Shyamal K. Talukder[1,2], Suresh Bhamidimarri[1,3], Konstantin Chekhovskiy[1] and Malay C. Saha[1]

[1]Noble Research Institute, LLC., 2510 Sam Noble Parkway, Ardmore, OK, USA

[2]California Cooperative Rice Research Foundation, Rice Expt. Station, 955 Butte city highway, Biggs, CA, USA

[3]Corteva Agriscience, 1040 Settler Rd., Connell, WA, USA

Supplementary figure and table

Supplementary Figure S1. Distribution of trait values under optimum growing condition in the growth chamber study. X-axis represents the trait values and Y-axis the number of genotypes. Traits name are written upright corner of the distribution.

Supplementary Figure S2. Linkage groups of R43-64 parental map. Total of 22 linkage groups were formed. Linkage group 8 and 21 has two component each shown as 8a, 8b and 21a and 21b, respectively.

Supplementary Figure S3. Linkage groups of 103-2 parental map. Total of 23 linkage groups were formed. Linkage group 2, 4, 6, 12 and 19 has two component each and shown as 2a, 2b, 4a, 4b, 6a, 6b, 12a, 12b, 19a and 19b, respectively. Linkage group 14 has three components indicated as 14a, 14b and 14c.

Supplementary Figure S4. Epistatic interaction among various QTL regions in female (103-2) parental linkage groups (LG) based on growth chamber study. Each circle indicates epistatic interaction for single trait, which is written in the center of each circle. Different color of the circle represents the interacting LGs. The LGs name with color legend are provided in the middle. The connecting line shows the interaction between the LGs. The number on the LGs denotes the centiMorgan (cM) position of interacting regions. The number on the connecting line represents LOD threshold of significance. The traits name outside the circle are the previously detected QTL position associated with those traits.

Supplementary Table S1. Expected mean squares of various phenotypic traits obtained from 103-2 × R43-64 tall fescue pseudo F_1 testcross mapping population evaluated in growth chamber and field experiments.

Supplementary Table S2. Correlation among the measured traits in the growth chamber experiment

Supplementary Table S3. Average trait value differences of the genotypes between optimum and summer dormant growing condition in growth chamber study. All abbreviated trait name starts with “D” that indicates difference value of the trait between optimum and summer dormant growing conditions.

Supplementary Figure S1

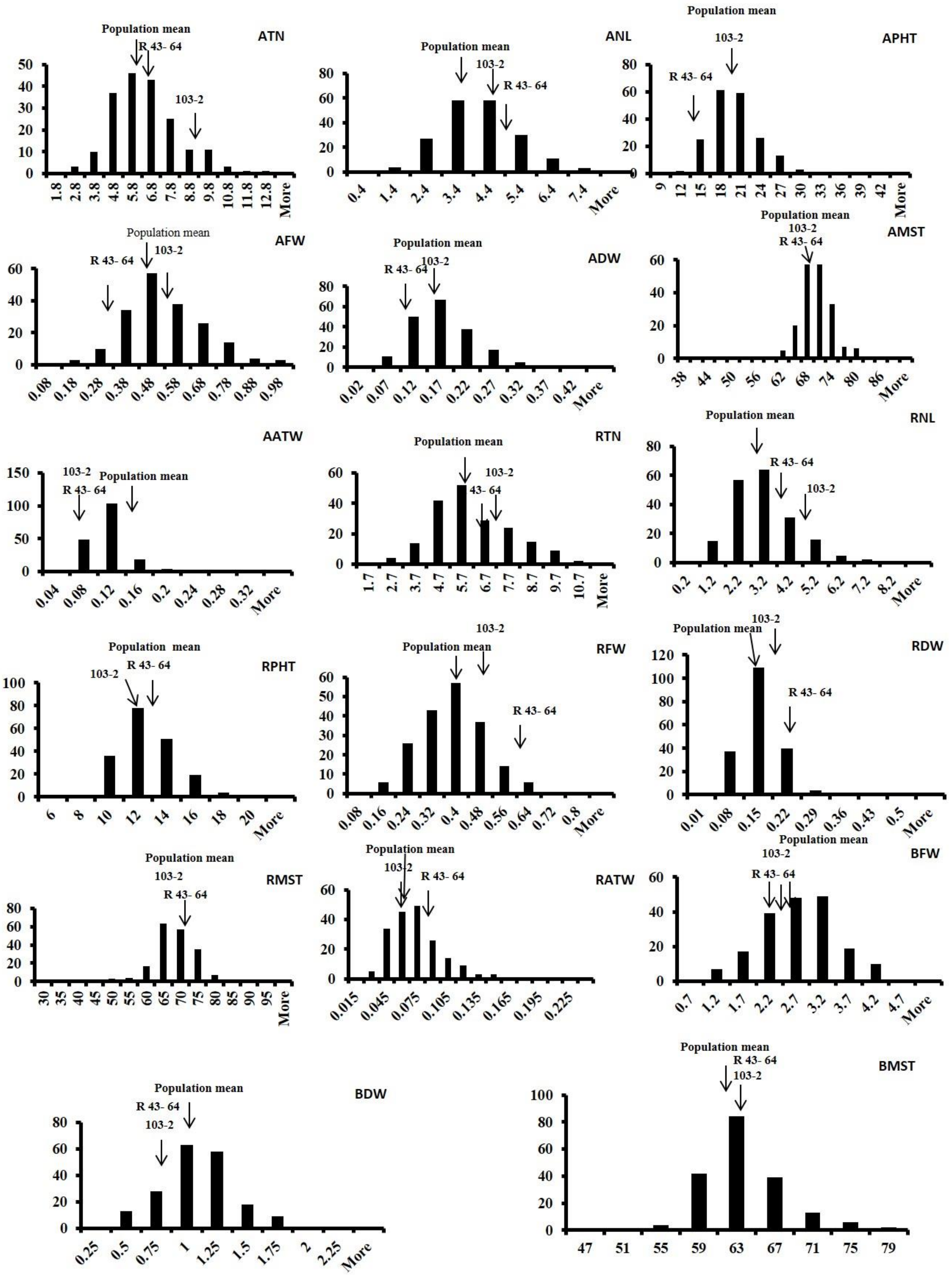

BFW- before cut back fresh weight; BDW- before cut back dry weight; BMST- before cut back moisture content; ATN- after cut back tiller number; ANL- after cut back new leaf; APHT- after cut back plant height; AFW- after cut back fresh weight; ADW- after cut back dry weight; AATW- after cut back average tiller weight; AMST- after cut back moisture content; RTN- return to normal growth tiller number; RNL- return to normal growth new leaf; RPHT- return to normal growth plant height; RFW- return to normal growth fresh weight; RDW- return to normal growth dry weight; RATW- return to normal growth average tiller weight; RMST- return to normal growth moisture content.

Supplementary Figure S2

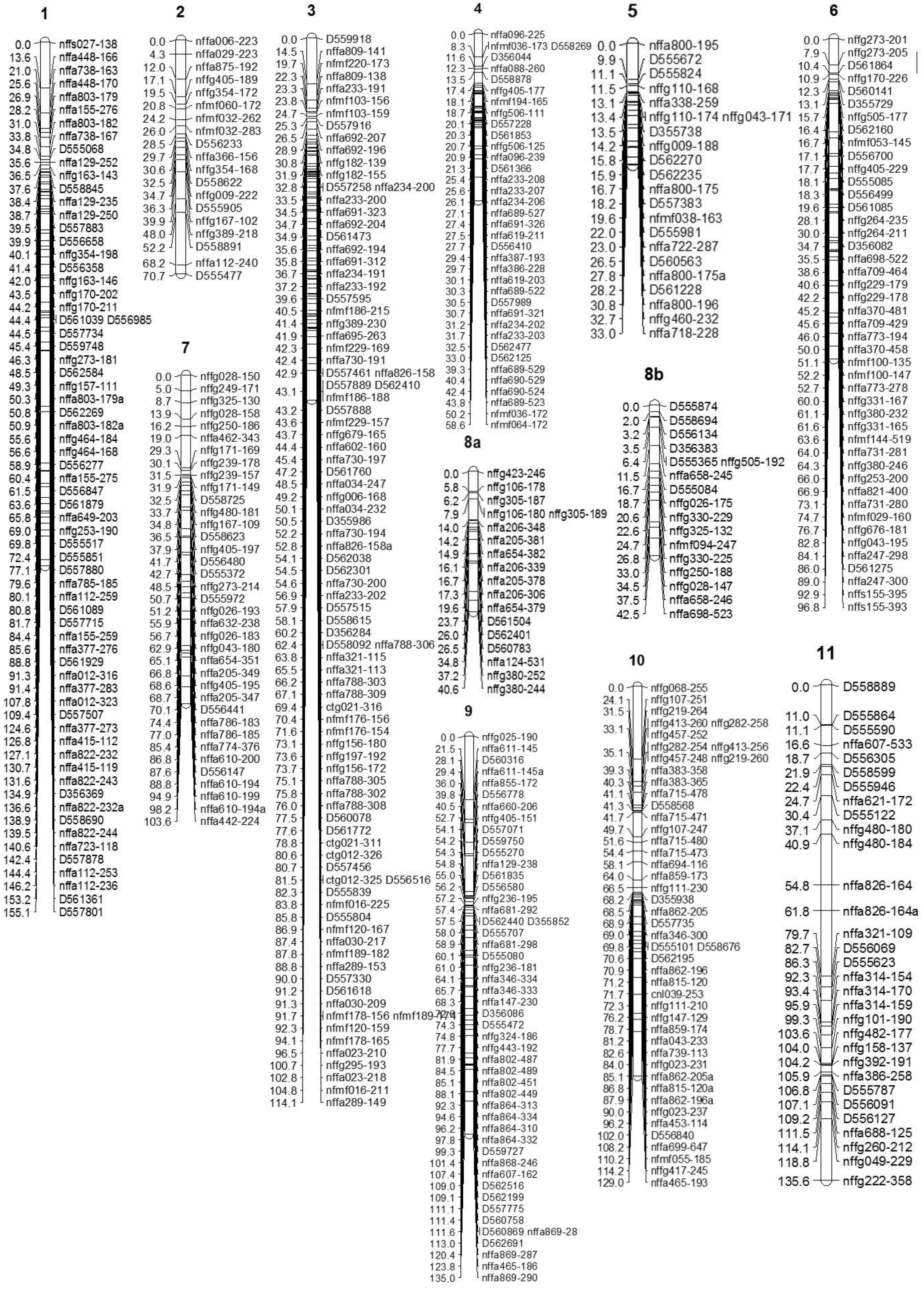

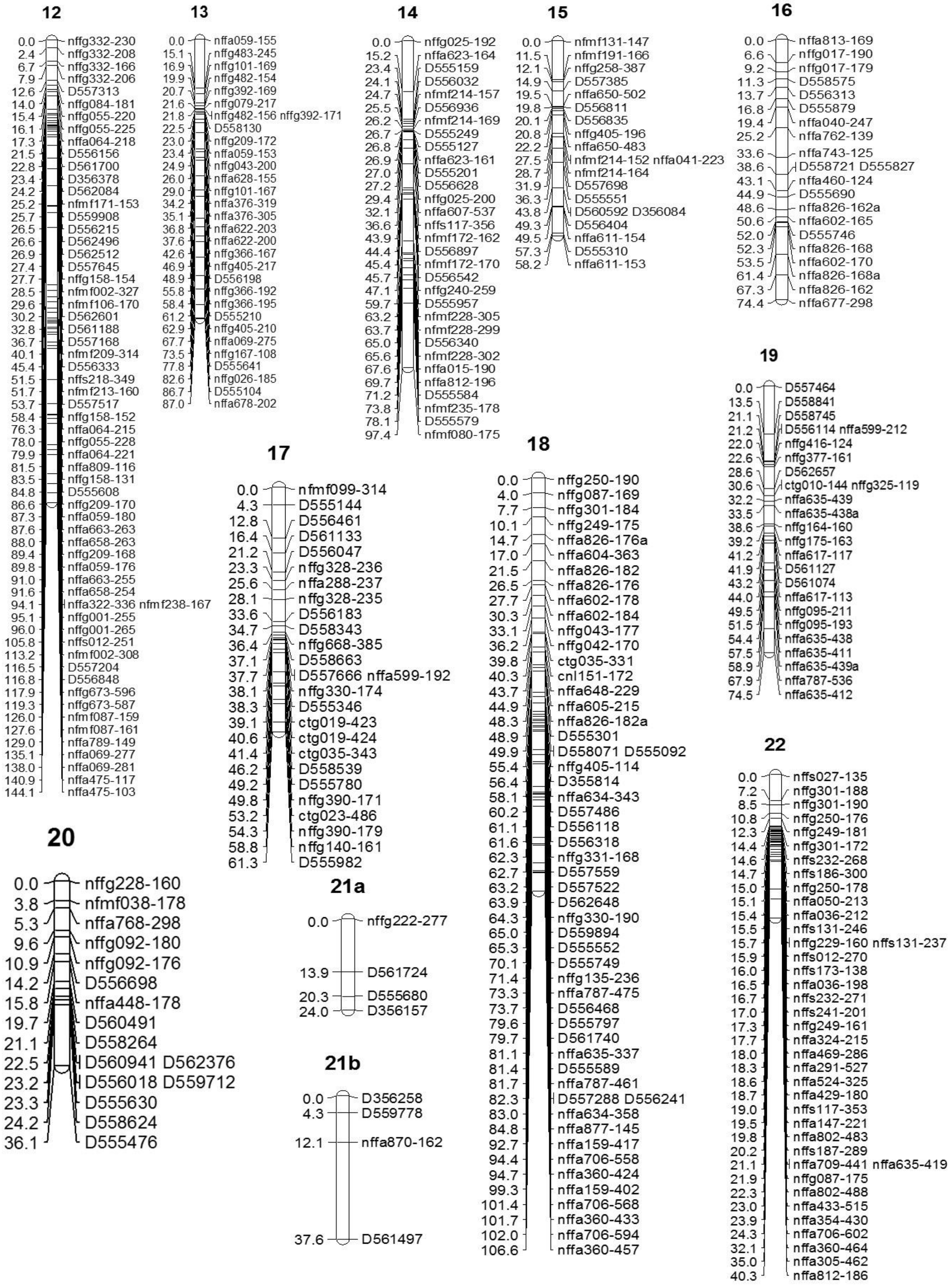
12
0.0 nffg332-230
2.4 nffg332-208
6.7 nffg332-166
7.9 nffg332-206
12.6 D557313
14.0 nffg084-181
15.4 nffg055-220
16.1 nffg055-225
17.3 nffa064-218
21.5 D556156
22.8 D561700
23.4 D356378
24.2 D562084
25.2 nfmf171-153
25.7 D559908
26.5 D556215
26.6 D562496
26.9 D562512
27.4 D557645
27.7 nffg158-154
28.5 nfmf002-327
29.6 nfmf106-170
30.2 D562601
32.8 D561188
36.7 D557168
40.1 nfmf209-314
45.4 D556333
51.5 nffs218-349
51.7 nfmf213-160
53.7 D557517
58.4 nffg158-152
76.3 nffa064-215
78.0 nffg055-228
79.9 nffa064-221
81.5 nffa809-116
83.5 nffg158-131
84.8 D555608
86.6 nffg209-170
87.3 nffa059-180
87.6 nffa663-263
88.0 nffa658-263
89.4 nffg209-168
89.8 nffa059-176
91.0 nffa663-255
91.6 nffa658-254
94.1 nffa322-336 nfmf238-167
95.1 nffg001-255
96.0 nffg001-265
105.8 nffs012-251
113.2 nfmf002-308
116.5 D557204
116.8 D556848
117.9 nffg673-596
119.3 nffg673-587
126.0 nfmf087-159
127.6 nfmf087-161
129.0 nffa789-149
135.1 nffa069-277
138.0 nffa069-281
140.9 nffa475-117
144.1 nffa475-103
13
0.0 nffa059-155
15.1 nffg483-245
16.9 nffg101-169
19.9 nffg482-154
20.7 nffg392-169
21.6 nffg079-217
21.8 nffg482-156 nffg392-171
22.5 D558130
23.0 nffg209-172
23.4 nffa059-153
24.9 nffg043-200
26.0 nffa628-155
29.0 nffg101-167
34.2 nffa376-319
35.1 nffa376-305
36.8 nffa622-203
37.6 nffa622-200
42.6 nffg366-167
46.9 nffg405-217
48.9 D556198
55.8 nffg366-192
58.4 nffg366-195
61.2 D555210
62.9 nffg405-210
67.7 nffa069-275
73.5 nffg167-108
77.8 D555641
82.6 nffg026-185
86.7 D555104
87.0 nffa678-202
14
0.0 nffg025-192
15.2 nffa623-164
23.4 D555159
24.1 D556032
24.7 nfmf214-157
25.5 D556936
26.2 nfmf214-169
26.7 D555249
26.8 D555127
26.9 nffa623-161
27.0 D555201
27.2 D556628
29.4 nffg025-200
32.1 nffa607-537
36.6 nffs117-356
43.9 nfmf172-162
44.4 D556897
45.4 nfmf172-170
45.7 D556542
47.1 nffg240-259
59.7 D555957
63.2 nfmf228-305
63.7 nfmf228-299
65.0 D556340
65.6 nfmf228-302
67.6 nffa015-190
69.7 nffa812-196
71.2 D555584
73.8 nfmf235-178
78.1 D555579
97.4 nfmf080-175
15
0.0 nfmf131-147
11.5 nfmf191-166
12.1 nffg258-387
14.9 D557385
19.5 nffa650-502
19.8 D556811
20.1 D556835
20.8 nffg405-196
22.2 nffa650-483
27.5 nfmf214-152 nffa041-223
28.7 nfmf214-164
31.9 D557698
36.3 D555551
43.8 D560592 D356084
49.3 D556404
49.5 nffa611-154
57.3 D555310
58.2 nffa611-153
16
0.0 nffa813-169
6.6 nffg017-190
9.2 nffg017-179
11.3 D558575
13.7 D556313
16.8 D555879
19.4 nffa040-247
25.2 nffa762-139
33.6 nffa743-125
38.6 D558721 D555827
43.1 nffa460-124
44.9 D555690
48.6 nffa826-162a
50.6 nffa602-165
52.0 D555746
52.3 nffa826-168
53.5 nffa602-170
61.4 nffa826-168a
67.3 nffa826-162
74.4 nffa677-298
19
0.0 D557464
13.5 D558841
21.1 D558745
21.2 D556114 nffa599-212
22.0 nffg416-124
22.6 nffg377-161
28.6 D562657
30.6 ctg010-144 nffg325-119
32.2 nffa635-439
33.5 nffa635-438a
38.6 nffg164-160
39.2 nffg175-163
41.2 nffa617-117
41.9 D561127
43.2 D561074
44.0 nffa617-113
49.5 nffg095-211
51.5 nffg095-193
54.4 nffa635-438
57.5 nffa635-411
58.9 nffa635-439a
67.9 nffa787-536
74.5 nffa635-412
17
0.0 nfmf099-314
4.3 D555144
12.8 D556461
16.4 D561133
21.2 D556047
23.3 nffg328-236
25.6 nffa288-237
28.1 nffg328-235
33.6 D556183
34.7 D558343
36.4 nffg668-385
37.1 D558663
37.7 D557666 nffa599-192
38.1 nffg330-174
38.3 D555346
39.1 ctg019-423
40.6 ctg019-424
41.4 ctg035-343
46.2 D558539
49.2 D555780
49.8 nffg390-171
53.2 ctg023-486
54.3 nffg390-179
58.8 nffg140-161
61.3 D555982
18
0.0 nffg250-190
4.0 nffg087-169
7.7 nffg301-184
10.1 nffg249-175
14.7 nffa826-176a
17.0 nffa604-363
21.5 nffa826-182
26.5 nffa826-176
27.7 nffa602-178
30.3 nffa602-184
33.1 nffg043-177
36.2 nffg042-170
39.8 ctg035-331
40.3 cnl151-172
43.7 nffa648-229
44.9 nffa605-215
48.3 nffa826-182a
48.9 D555301
49.9 D558071 D555092
55.4 nffg405-114
56.4 D355814
58.1 nffa634-343
60.2 D557486
61.1 D556118
61.6 D556318
62.3 nffg331-168
62.7 D557559
63.2 D557522
63.9 D562648
64.3 nffg330-190
65.0 D559894
65.3 D555552
70.1 D555749
71.4 nffg135-236
73.3 nffa787-475
73.7 D556468
79.6 D555797
79.7 D561740
81.1 nffa635-337
81.4 D555589
81.7 nffa787-461
82.3 D557288 D556241
83.0 nffa634-358
84.8 nffa877-145
92.7 nffa159-417
94.4 nffa706-558
94.7 nffa360-424
99.3 nffa159-402
101.4 nffa706-568
101.7 nffa360-433
102.0 nffa706-594
106.6 nffa360-457
20
0.0 nffg228-160
3.8 nfmf038-178
5.3 nffa768-298
9.6 nffg092-180
10.9 nffg092-176
14.2 D556698
15.8 nffa448-178
19.7 D560491
21.1 D558264
22.5 D560941 D562376
23.2 D556018 D559712
23.3 D555630
24.2 D558624
36.1 D555476
21a
0.0 nffg222-277
13.9 D561724
20.3 D555680
24.0 D356157
21b
0.0 D356258
4.3 D559778
12.1 nffa870-162
37.6 D561497
22
0.0 nffs027-135
7.2 nffg301-188
8.5 nffg301-190
10.8 nffg250-176
12.3 nffg249-181
14.4 nffg301-172
14.6 nffs232-268
14.7 nffs186-300
15.0 nffg250-178
15.1 nffa050-213
15.4 nffa036-212
15.5 nffs131-246
15.7 nffg229-160 nffs131-237
15.9 nffs012-270
16.0 nffs173-138
16.5 nffa036-198
16.7 nffs232-271
17.0 nffs241-201
17.3 nffg249-161
17.7 nffa324-215
18.0 nffa469-286
18.3 nffa291-527
18.6 nffa524-325
18.7 nffa429-180
19.0 nffs117-353
19.5 nffa147-221
19.8 nffa802-483
20.2 nffs187-289
21.1 nffa709-441 nffa635-419
21.9 nffg087-175
22.3 nffa802-488
23.0 nffa433-515
23.9 nffa354-430
24.3 nffa706-602
32.1 nffa360-464
35.0 nffa305-462
40.3 nffa812-186

Supplementary Figure S3

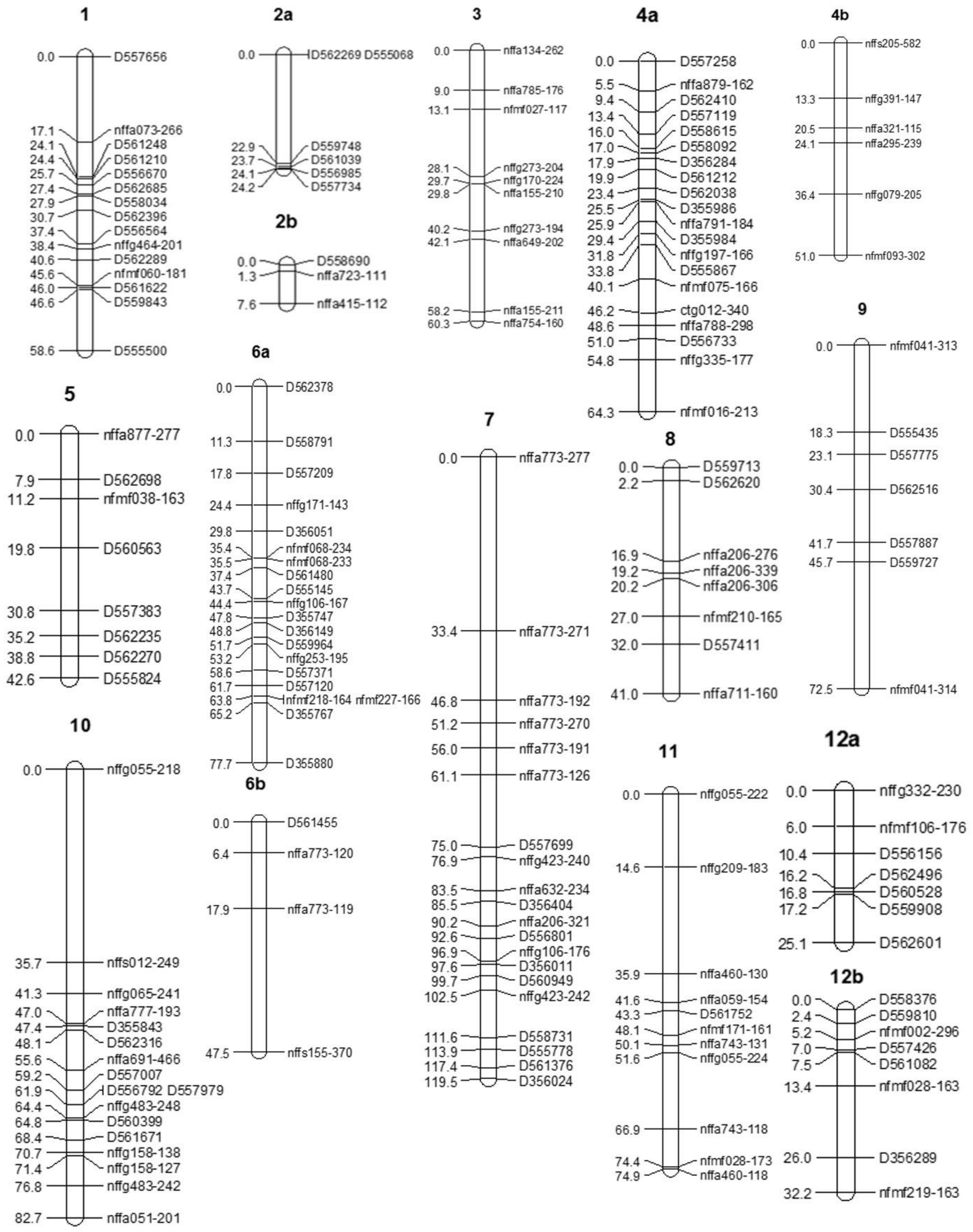

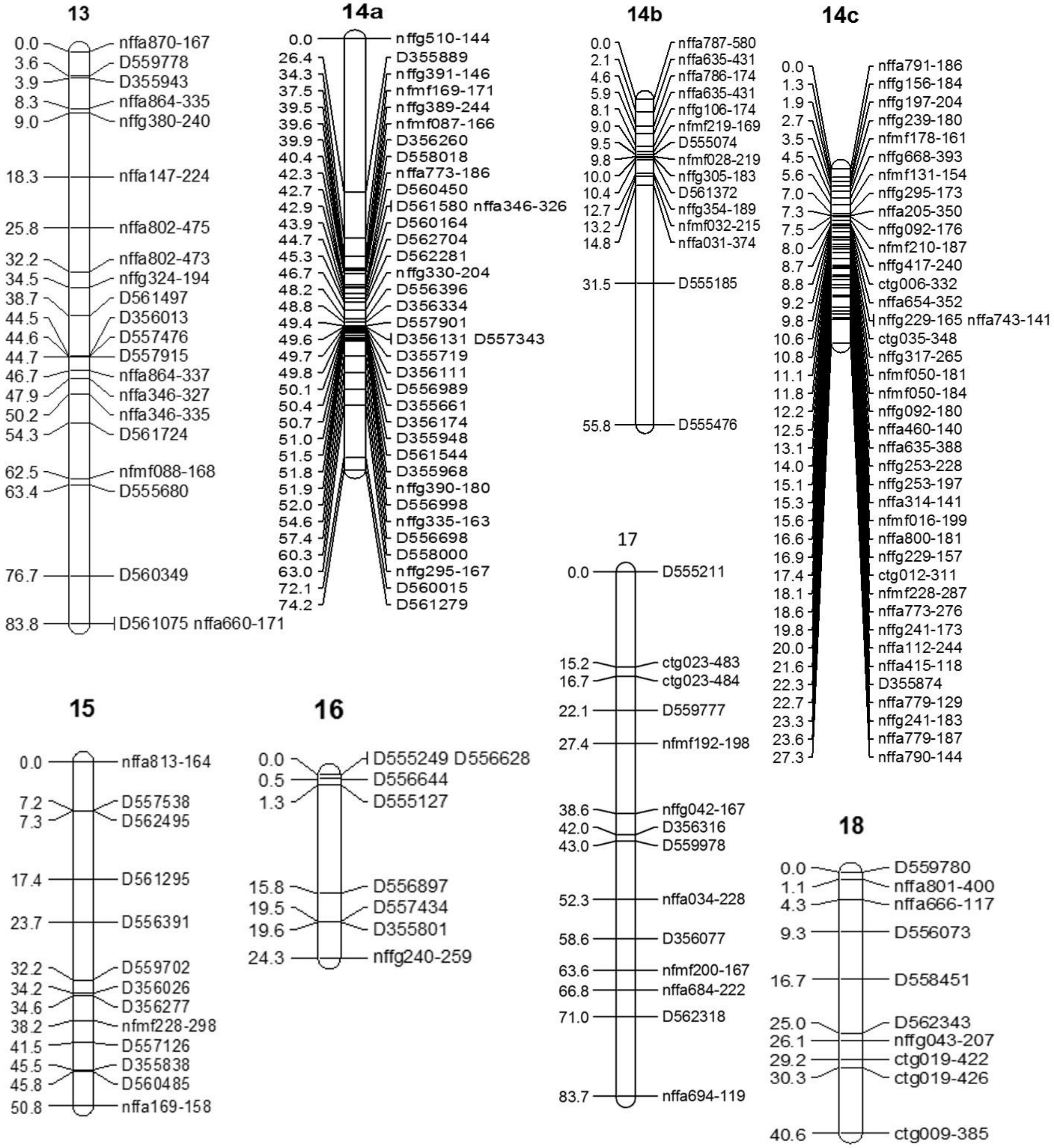
13
0.0 nffa870-167
3.6 D559778
3.9 D355943
8.3 nffa864-335
9.0 nffg380-240
18.3 nffa147-224
25.8 nffa802-475
32.2 nffa802-473
34.5 nffg324-194
38.7 D561497
44.5 D356013
44.6 D557476
44.7 D557915
46.7 nffa864-337
47.9 nffa346-327
50.2 nffa346-335
54.3 D561724
62.5 nfmf088-168
63.4 D555680
76.7 D560349
83.8 D561075 nffa660-171
14a
0.0 nffg510-144
26.4 D355889
34.3 nffg391-146
37.5 nfmf169-171
39.5 nffg389-244
39.6 nfmf087-166
39.9 D356260
40.4 D558018
42.3 nffa773-186
42.7 D560450
42.9 D561580 nffa346-326
43.9 D560164
44.7 D562704
45.3 D562281
46.7 nffg330-204
48.2 D556396
48.8 D356334
49.4 D557901
49.6 D356131 D557343
49.7 D355719
49.8 D356111
50.1 D556989
50.4 D355661
50.7 D356174
51.0 D355948
51.5 D561544
51.8 D355968
51.9 nffg390-180
52.0 D556998
54.6 nffg335-163
57.4 D556698
60.3 D558000
63.0 nffg295-167
72.1 D560015
74.2 D561279
14b
0.0 nffa787-580
2.1 nffa635-431
4.6 nffa786-174
5.9 nffa635-431
8.1 nffg106-174
9.0 nfmf219-169
9.5 D555074
9.8 nfmf028-219
10.0 nffg305-183
10.4 D561372
12.7 nffg354-189
13.2 nfmf032-215
14.8 nffa031-374
31.5 D555185
55.8 D555476
14c
0.0 nffa791-186
1.3 nffg156-184
1.9 nffg197-204
2.7 nffg239-180
3.5 nfmf178-161
4.5 nffg668-393
5.6 nfmf131-154
7.0 nffg295-173
7.3 nffa205-350
7.5 nffg092-176
8.0 nfmf210-187
8.7 nffg417-240
8.8 ctg006-332
9.2 nffa654-352
9.8 nffg229-165 nffa743-141
10.6 ctg035-348
10.8 nffg317-265
11.1 nfmf050-181
11.8 nfmf050-184
12.2 nffg092-180
12.5 nffa460-140
13.1 nffa635-388
14.0 nffg253-228
15.1 nffg253-197
15.3 nffa314-141
15.6 nfmf016-199
16.6 nffa800-181
16.9 nffg229-157
17.4 ctg012-311
18.1 nfmf228-287
18.6 nffa773-276
19.8 nffg241-173
20.0 nffa112-244
21.6 nffa415-118
22.3 D355874
22.7 nffa779-129
23.3 nffg241-183
23.6 nffa779-187
27.3 nffa790-144
15
0.0 nffa813-164
7.2 D557538
7.3 D562495
17.4 D561295
23.7 D556391
32.2 D559702
34.2 D356026
34.6 D356277
38.2 nfmf228-298
41.5 D557126
45.5 D355838
45.8 D560485
50.8 nffa169-158
16
0.0 D555249 D556628
0.5 D556644
1.3 D555127
15.8 D556897
19.5 D557434
19.6 D355801
24.3 nffg240-259
17
0.0 D555211
15.2 ctg023-483
16.7 ctg023-484
22.1 D559777
27.4 nfmf192-198
38.6 nffg042-167
42.0 D356316
43.0 D559978
52.3 nffa034-228
58.6 D356077
63.6 nfmf200-167
66.8 nffa684-222
71.0 D562318
83.7 nffa694-119
18
0.0 D559780
1.1 nffa801-400
4.3 nffa666-117
9.3 D556073
16.7 D558451
25.0 D562343
26.1 nffg043-207
29.2 ctg019-422
30.3 ctg019-426
40.6 ctg009-385

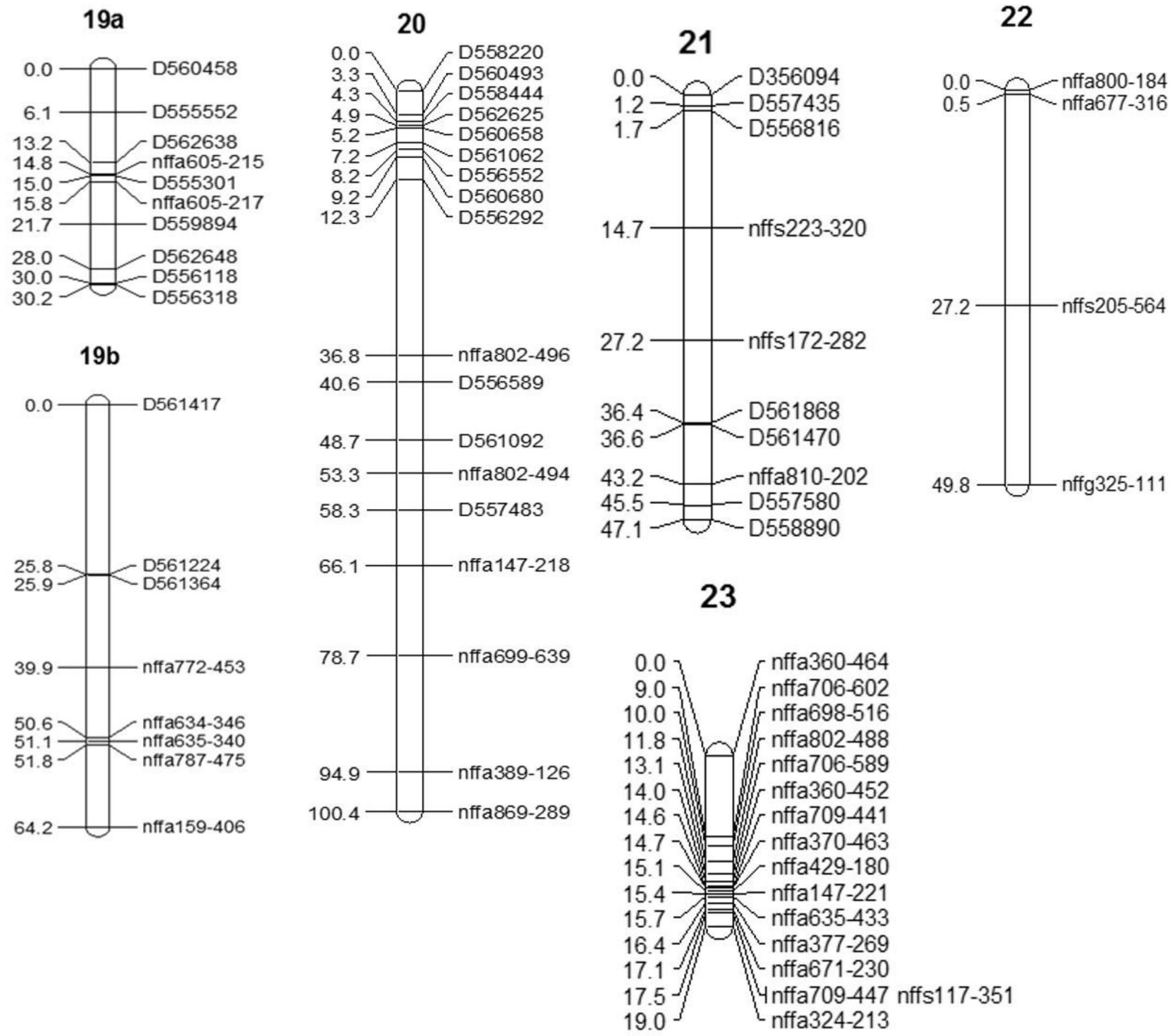
19a
0.0 D560458
6.1 D555552
13.2 D562638
14.8 nffa605-215
15.0 D555301
15.8 nffa605-217
21.7 D559894
28.0 D562648
30.0 D556118
30.2 D556318
19b
0.0 D561417
25.8 D561224
25.9 D561364
39.9 nffa772-453
50.6 nffa634-346
51.1 nffa635-340
51.8 nffa787-475
64.2 nffa159-406
20
0.0 D558220
3.3 D560493
4.3 D558444
4.9 D562625
5.2 D560658
7.2 D561062
8.2 D556552
9.2 D560680
12.3 D556292
36.8 nffa802-496
40.6 D556589
48.7 D561092
53.3 nffa802-494
58.3 D557483
66.1 nffa147-218
78.7 nffa699-639
94.9 nffa389-126
100.4 nffa869-289
21
0.0 D356094
1.2 D557435
1.7 D556816
14.7 nffs223-320
27.2 nffs172-282
36.4 D561868
36.6 D561470
43.2 nffa810-202
45.5 D557580
47.1 D558890
22
0.0 nffa800-184
0.5 nffa677-316
27.2 nffs205-564
49.8 nffg325-111
23
0.0 nffa360-464
9.0 nffa706-602
10.0 nffa698-516
11.8 nffa802-488
13.1 nffa706-589
14.0 nffa360-452
14.6 nffa709-441
14.7 nffa370-463
15.1 nffa429-180
15.4 nffa147-221
15.7 nffa635-433
16.4 nffa377-269
17.1 nffa671-230
17.5 nffa709-447 nffs117-351
19.0 nffa324-213

Supplementary Figure S4

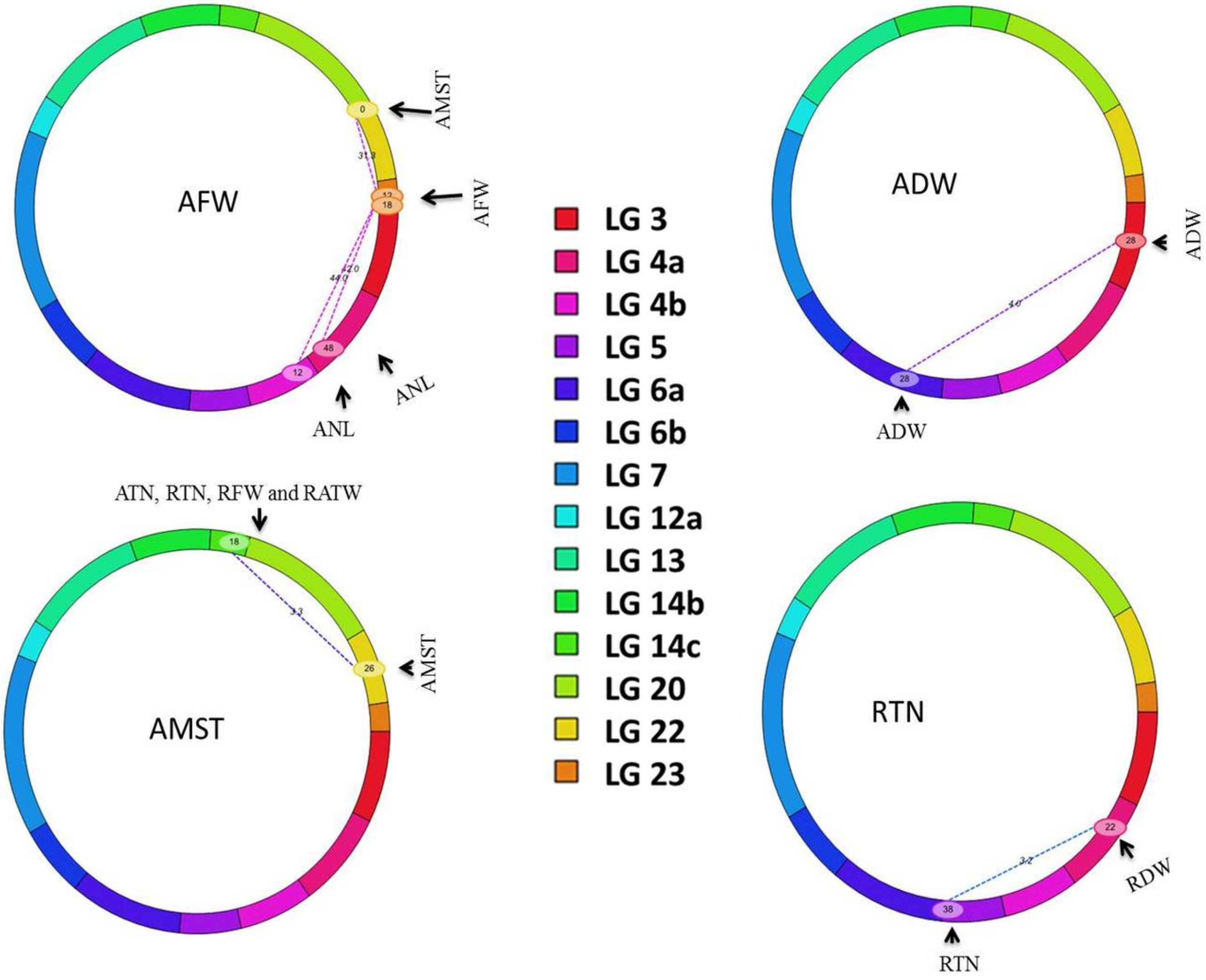

AFW- after cut back fresh weight; ADW- after cut back dry weight; AMST- after cut back moisture content; RTN- return to normal growth tiller number; ANL- after cut back new leaf and RDW- return to normal growth dry weight

Table S1 Expected mean squares of various phenotypic traits obtained from 103-2 × R43-64 tall fescue pseudo F1 testcross mapping population evaluated in growth chamber and field experiments.

A. growth chamber experiment

Source	DF	ATN	ANL	APHT	AFW	ADW	AMST	AATW	RTN
Rep	1	5.53	4.4	138.76**	64.61*	2.289**	2098.25**	1.98**	57.19
Gen	191	10.36**	4.53**	38.13**	7.85	0.012**	170.97	0.31	10.25
Trt	1	210.47**	39.62**	3360.84**	1.95	0.05**	2879.12**	0.24	276.21
Gen*Trt	190	2.9	2.25	10.76	7.78	0.005	141.33	0.31	2.56

Source	RNL	RPHT	RFW	RDW	RMST	RATW	BFW	BDW	BMST
Rep	4.67	74.15**	1.97**	0.65**	9518.55**	0.11**	17.39**	0.63**	3030.80**
Gen	5.87**	13.94**	0.03	0.006**	149.01	0.002*	1.86**	0.35**	40.55
Trt	162.86**	54.96**	0.03	0.03**	2889.56**	0.02**	3.09**	3.18**	11682.84**
Gen*Trt	2.45	4.08	0.02	0.004	132.6	0.001	0.3	0.07	29.49

B. Field experiments

Source	DF	SPQ	SPHT	SFW	SMST	SDW	FPHT	FFW	FDW	FMST
Gen	192	3.96**	244.45	100192.61**	62.66**	20556.76**	19.64**	32435.36**	4335.25**	125.06
Loc	1	105.62**	3.16	3218.66	1.23	205.21	1232.24**	2562634	506330.69**	3802.37**
Gen*Loc	4	61.5**	1061.82**	498306.81**	439.44**	90911.09**	76.80*	237686.71**	16576.65**	1470.05**

DF- degrees of freedom; Rep- Replication; Gen- Genotype; Trt-Treatment; Loc- Location; ATN- after cut back tiller number; ANL- after cut back new leaf; APHT- after cut back plant height; AFW- after cut back fresh weight; ADW- after cut back dry weight; AMST- after cut back moisture content; AATW- after cut back average tiller weight; RTN- return to normal growth tiller number; RNL- return to normal growth new leaf; RPHT- return to normal growth plant height; RFW- return to normal growth fresh weight; RDW- return to normal growth dry weight; RMST- return to normal growth moisture content; RATW-return to normal growth average tiller weight; BFW- before cut back fresh weight; BDW- before cut back dry weight; BMST- before cut back moisture content;. SPQ- spring plant quality; SPHT- spring plant height; SFW- spring fresh biomass weight; SMST- spring plant moisture content; SDW- spring dry biomass weight; FPHT- fall plant height; FFW- fall fresh biomass weight; FDW- fall dry biomass weight; FMST- fall plant moisture content.

*p ≤ 0.05; **p ≤ 0.01

Table S2 Correlation among the measured traits in the growth chamber experiment

	N†	ATN	ANL	APHT	AFW	ADW	AMST	AATW	RTN	RNL	RPHT	RFW	RDW	RMST	RATW	BFW	BDW
ATN	765																
ANL	765	0.59**															
APHT	765	-0.27**	-0.19**														
AFW	761	-0.02	0.016	0.039													
ADW	763	0.07	0.09**	0.24**	0.05												
AMST	760	-0.05	-0.09**	0.15**	0.08*	-0.10**											
AATW	760	-0.15**	0.10**	-0.13**	0.46**	0.24**	-0.25**										
RTN	762	0.84**	0.49**	-0.26**	-0.03	0.09**	-0.014	-0.19**									
RNL	762	0.48**	0.30**	-0.25**	-0.03	0.02	-0.07*	0.09*	0.59**								
RPHT	762	-0.28**	-0.27**	0.46**	0.002	0.04	0.09**	-0.19**	-0.28**	-0.25**							
RFW	710	-0.13**	0.12**	-0.15**	0.06	0.26**	-0.32**	0.88**	-0.18**	0.12**	-0.15**						
RDW	710	0.07*	0.12**	-0.017	0.02	-0.01	-0.15**	0.29**	0.041	0.10**	0.19**	0.48**					
RMST	710	0.0002	-0.06	0.24**	0.001	0.09*	0.37**	-0.31**	0.004	-0.08*	0.10**	-0.32**	-0.38**				
RATW	710	-0.16**	0.11**	*0.18**	0.06	0.25**	-0.35**	0.91**	-0.20**	0.12**	-0.23**	0.97**	0.33**	-0.35**			
BFW	755	0.24**	0.10**	0.41**	0.03	0.42**	0.10**	-0.16**	0.26**	-0.03	0.28**	-0.14**	0.14**	0.16**	-0.21**		
BDW	757	0.21**	0.17**	0.21**	0.04	0.46**	0.06*	0.17**	0.22**	0.06	0.18**	0.24**	0.32**	-0.02	0.18**	0.84**	
BMST	754	0.03	-0.09*	0.30**	0.015*	-0.06	0.24**	-0.44**	0.05	-0.11**	0.12**	-0.53**	-0.27**	0.24**	-0.52**	0.18**	-0.30**

ATN- after cut back tiller number; ANL- after cut back new leaf; APHT- after cut back plant height; AFW- after cut back fresh weight; ADW- after cut back dry weight; AMST- after cut back moisture content; AATW- after cut back average tiller weight; RTN- return to normal growth tiller number; RNL- return to normal growth new leaf; RPHT- return to normal growth plant height; RFW- return to normal growth fresh weight; RDW- return to normal growth dry weight; RMST- return to normal growth moisture content; RATW-return to normal growth average tiller weight; BFW- before cut back fresh weight; BDW- before cut back dry weight.

†N- Number of observations used to measure the correlation

*$p \leq 0.05$; **$p \leq 0.01$

Table S3 Average trait value differences of the genotypes between optimum and summer dormant growing conditions in growth chamber study. The letter "D" prefixed every trait name indicates difference between trait values at optimum and summer dormant growing conditions.

Genotype	DBFW	DBDW	DBMST	DATN	DANL	DAPHT	DAFW	DADW	DAMST	DAATW	DRTN	DRNL	DRPHT	DRFW	DRDW	DRMST	DRATW
Genotype 1	-0.86	-0.10	-10.23	1.50	-0.50	-4.50	-0.25	-0.05	-11.25	-0.05	-0.50	-1.00	0.50	-0.10	-0.02	-21.31	-0.012
Genotype 2	-0.69	0.08	-9.90	2.50	1.00	-5.50	-0.10	-0.05	-9.05	-0.04	5.50	5.50	-2.75	0.18	0.07	0.23	-0.007
Genotype 3	-0.16	0.19	-5.77	-1.00	-2.00	6.00	0.50	0.15	0.30	0.07	0.00	0.50	2.00	0.12	0.08	-2.02	0.013
Genotype 4	-0.28	0.21	-9.36	2.00	0.50	-6.00	0.25	0.05	0.95	0.01	2.00	0.50	1.40	0.01	0.00	1.53	-0.012
Genotype 5	-0.22	0.25	-9.84	-1.50	-1.00	-6.75	0.35	0.15	-3.40	0.06	-1.00	0.00	0.85	0.00	-0.07	16.67	0.007
Genotype 6	-0.80	-0.18	-4.23	0.50	1.50	-9.75	0.20	0.10	-4.15	0.03	1.00	0.00	1.25	0.21	0.08	5.85	0.024
Genotype 9	-1.34	-0.42	-4.01	1.00	0.50	-4.75	-0.20	-0.10	-6.65	-0.03	1.50	3.50	-2.35	-0.36	-0.16	-5.60	-0.043
Genotype 10	-0.85	-0.24	-4.15	-1.00	0.00	-5.25	-0.05	-0.05	1.80	0.01	0.50	0.00	-2.15	-0.09	-0.03	-2.09	-0.020
Genotype 11	0.00	0.16	-5.12	0.00	2.00	-3.50	0.30	0.15	-13.70	0.06	1.00	-1.00	2.65	0.05	0.03	-11.29	-0.003
Genotype 14	-0.83	-0.19	-5.21	-1.00	0.00	-9.00	0.15	0.10	1.05	0.07	-1.00	-1.50	1.90	0.07	0.06	-6.78	0.034
Genotype 15	-1.64	-0.58	-4.36	0.00	0.00	-3.50	0.30	0.15	-13.45	0.05	0.00	0.50	2.15	-0.09	0.01	-11.24	-0.013
Genotype 16	-1.68	-0.54	-7.52	-2.00	-1.50	-2.50	-0.05	-0.05	0.80	0.02	-2.00	-0.50	-2.10	-0.04	0.01	-7.60	0.018
Genotype 17	-0.39	0.01	-7.60	2.00	1.00	-2.50	-0.15	-0.05	-7.65	-0.04	1.00	2.50	-0.50	0.04	0.07	-28.40	0.000
Genotype 18	-0.29	0.08	-12.88	5.00	1.50	-3.00	-0.20	-0.05	-9.90	-0.06	5.00	3.50	-2.10	-0.02	0.02	-27.64	-0.029
Genotype 19	-0.64	-0.14	-6.17	0.50	2.00	-2.50	0.50	0.00	18.40	0.06	0.00	0.50	-2.25	-0.08	-0.01	-9.25	-0.011
Genotype 20	-0.03	0.25	-18.63	1.50	2.00	-1.50	0.00	0.00	-7.60	-0.01	1.00	0.00	0.25	-0.03	-0.02	8.81	-0.011
Genotype 21	-0.29	0.16	-10.16	1.00	0.00	-8.75	-0.10	-0.10	6.00	-0.02	-1.50	-1.50	-1.45	0.23	0.04	2.34	0.035
Genotype 22	0.43	0.11	0.54	1.50	0.50	-2.25	0.15	0.00	24.25	0.01	0.00	0.00	-1.50	0.01	0.04	-12.45	0.001
Genotype 24	-0.21	0.12	-13.55	-2.00	-1.50	-2.75	0.20	0.10	6.55	0.04	-1.00	-2.00	-0.20	0.05	0.01	4.58	0.011
Genotype 25	0.86	0.58	-10.06	1.50	0.50	-6.50	-0.05	0.00	-10.50	-0.03	3.50	3.00	-6.40	-0.08	-0.03	3.18	-0.042
Genotype 26	-0.23	0.24	-12.07	3.00	2.00	-8.00	-0.05	0.00	-7.50	-0.06	4.00	1.00	-2.20	-0.01	0.02	-6.57	-0.036
Genotype 27	0.12	-0.01	2.77	1.50	0.50	-7.50	0.05	-0.05	8.80	-0.01	2.00	0.50	0.00	-0.01	0.02	-8.82	-0.012
Genotype 28	-0.34	-0.05	-3.05	1.00	0.50	-5.75	0.30	0.10	-3.85	0.03	1.00	1.00	1.90	0.06	0.04	-7.29	0.000
Genotype 29	-0.42	0.24	-20.12	-1.00	-1.00	-8.00	-0.20	0.00	-6.20	-0.02	0.50	-1.00	-2.40	-0.14	-0.03	-6.37	-0.028
Genotype 30	0.06	0.29	-13.63	4.00	0.00	-5.50	0.00	0.00	-10.20	-0.02	3.00	0.50	-2.10	-0.15	-0.01	-12.06	-0.027
Genotype 31	0.25	0.16	-2.57	2.50	0.50	-8.25	0.00	0.00	5.60	-0.02	2.50	0.50	-1.65	0.09	0.06	-7.80	-0.002
Genotype 32	0.22	0.22	-4.11	1.00	1.50	-5.50	-0.10	0.00	-6.55	-0.03	2.50	1.00	-2.20	-0.03	0.02	-3.85	-0.032
Genotype 33	-0.46	0.05	-10.39	-1.00	-1.50	-2.50	0.05	0.05	-14.65	0.02	0.00	2.00	-2.45	0.13	0.08	-7.13	0.025
Genotype 34	0.22	0.28	-8.48	0.00	-2.00	-0.50	0.25	0.05	-13.25	0.05	0.50	0.50	3.45	0.05	0.03	-7.07	0.004
Genotype 36	0.14	0.27	-9.73	1.00	-0.50	-1.25	0.20	0.05	4.85	0.02	-0.50	-0.50	-2.05	-0.09	-0.04	1.54	-0.007
Genotype 37	-0.51	0.05	-9.51	0.00	-1.00	-5.50	-0.15	-0.05	-20.40	-0.02	-0.50	2.50	2.45	0.02	0.04	-10.62	0.007
Genotype 38	0.24	0.25	-4.63	3.50	2.50	-5.75	-0.15	0.05	13.40	-0.06	2.00	2.50	-0.40	-0.14	-0.04	-1.74	-0.048
Genotype 40	0.56	0.31	-2.60	3.50	2.50	-1.50	0.15	0.15	-24.10	-0.02	2.50	1.00	-3.00	-0.09	0.02	-32.45	-0.034
Genotype 41	-0.12	0.20	-9.69	5.00	1.00	-3.75	0.40	0.15	-9.85	0.01	2.50	1.00	-0.70	-0.02	0.10	-25.29	-0.029
Genotype 42	-0.27	0.03	-6.03	1.00	0.50	-4.25	0.15	0.10	-25.45	0.01	1.50	0.00	0.45	-0.03	0.00	-3.85	-0.012
Genotype 43	0.06	0.21	-8.65	3.00	-1.00	-6.50	0.15	0.05	8.30	-0.02	2.50	1.50	-2.15	-0.15	-0.03	-10.47	-0.042
Genotype 44	-0.28	-0.18	1.96	0.00	-1.00	-4.50	-0.10	0.05	-24.75	-0.02	-0.50	2.50	1.65	-0.13	0.01	-25.25	-0.020
Genotype 45	-0.61	-0.20	-1.59	1.50	2.00	-7.25	-0.35	-0.10	-24.10	-0.10	1.50	0.00	-0.25	-0.09	0.04	-30.72	-0.036
Genotype 46	0.14	0.24	-6.58	2.50	1.50	-3.50	-0.30	-0.05	-27.60	-0.07	2.50	3.00	0.00	-0.16	-0.02	-19.32	-0.045
Genotype 47	1.38	0.77	-6.74	2.50	1.50	-4.50	0.30	0.00	9.10	0.00	2.00	1.00	2.60	0.11	0.07	2.34	-0.021
Genotype 49	1.07	0.74	-10.45	1.50	1.00	-0.50	0.50	0.10	3.20	0.06	2.00	1.50	1.50	0.28	0.15	-8.89	0.016
Genotype 50	0.48	0.50	-11.82	2.50	0.50	-2.25	0.45	0.10	-8.55	0.03	2.00	1.50	1.75	0.02	0.00	1.50	-0.022
Genotype 51	-1.28	-0.38	-7.80	-0.50	0.00	-8.00	0.05	-0.05	2.70	0.03	-1.00	-0.50	-2.75	-0.11	-0.04	0.30	-0.010
Genotype 52	-0.43	0.02	-9.08	1.50	0.00	1.00	0.25	0.05	-26.05	0.01	0.50	0.50	5.00	0.19	0.09	-7.52	0.029
Genotype 54	-0.23	0.02	-3.69	1.50	-0.50	-7.25	0.25	0.05	3.15	-0.01	1.00	0.50	0.95	-0.06	-0.02	0.55	-0.036

Genotype 55	1.17	0.73	-6.71	1.50	1.50	0.00	0.15	0.05	-14.60	0.00	1.50	-0.50	-0.55	0.20	0.06	1.76	0.019
Genotype 57	1.03	0.77	-10.72	3.00	3.00	-3.50	0.60	0.20	-21.40	0.03	2.50	2.00	-1.40	-0.05	-0.04	4.17	-0.038
Genotype 58	-0.40	0.19	-9.12	2.50	2.00	-7.25	0.25	0.00	-8.15	-0.01	2.00	1.50	-3.50	-0.19	-0.08	5.06	-0.057
Genotype 59	0.29	0.52	-9.54	1.50	-1.50	-1.75	0.00	-0.05	0.00	-0.02	0.50	1.00	0.15	-0.25	-0.09	-9.07	-0.029
Genotype 60	-0.13	0.00	-3.05	4.50	2.00	-6.50	0.15	-0.10	-6.80	-0.06	4.00	2.50	-2.95	-0.06	-0.02	11.99	-0.029
Genotype 61	-1.05	-0.43	6.78	-1.00	-1.50	-7.25	0.15	-0.10	19.50	0.08	-1.00	-0.50	-2.65	0.03	-0.01	11.00	0.023
Genotype 62	-0.64	-0.24	-1.95	-0.50	-1.00	-3.00	0.30	0.00	17.60	0.05	-1.00	-1.00	0.55	-0.05	-0.04	9.19	-0.001
Genotype 63	-0.54	0.01	-8.45	2.50	0.50	-3.00	0.10	-0.05	1.40	-0.05	2.00	-0.50	0.00	0.02	0.02	-2.48	-0.035
Genotype 64	0.52	0.36	-5.35	2.50	2.00	-7.25	0.15	0.00	-2.90	-0.02	0.50	-1.50	-2.75	0.14	0.07	-7.52	0.023
Genotype 65	-0.08	0.06	-6.30	3.00	2.00	-2.25	0.35	0.05	1.30	0.01	1.50	-2.50	0.35	-0.01	-0.01	17.18	-0.013
Genotype 67	0.36	0.19	3.52	0.50	0.00	-2.00	0.30	0.05	1.65	0.07	1.00	1.00	-3.15	0.00	-0.05	29.09	-0.018
Genotype 68	-0.57	-0.23	-1.05	1.00	-0.50	-9.50	0.30	0.00	21.20	0.04	-0.50	0.50	-1.00	-0.08	-0.05	10.48	-0.011
Genotype 69	-0.18	-0.03	-3.20	1.50	2.00	-4.50	0.40	0.05	15.75	0.04	1.50	1.50	-2.30	0.07	0.00	17.29	-0.010
Genotype 70	0.28	0.24	-4.25	-1.00	-1.00	-0.50	0.15	-0.05	-1.85	0.04	0.00	1.00	-1.00	0.09	0.03	-0.60	0.014
Genotype 71	-0.86	-0.46	6.11	3.50	0.50	-3.00	0.45	0.05	-13.75	0.04	3.50	3.00	-1.60	0.08	0.05	-2.75	-0.013
Genotype 72	-0.90	-0.19	-12.36	0.00	-1.00	-3.25	0.20	0.05	-26.80	0.06	0.50	0.50	-2.00	0.04	0.04	-6.65	-0.002
Genotype 73	-0.13	0.09	-4.54	2.00	0.00	-1.50	0.40	0.10	4.95	0.03	2.50	3.00	-0.10	-0.06	-0.02	0.14	-0.036
Genotype 74	-0.87	-0.12	-12.16	1.50	0.50	-4.50	0.55	0.15	11.80	0.07	1.50	0.50	-0.75	0.17	0.03	16.23	0.017
Genotype 76	-0.15	0.06	-4.80	-2.00	-2.50	0.00	0.70	0.20	5.80	0.15	-2.00	-1.50	2.50	0.11	0.10	-11.48	0.030
Genotype 77	-0.56	-0.12	-5.90	1.00	-1.50	-5.00	0.35	0.10	-10.90	0.03	-0.50	-0.50	-2.35	.	.	.	.
Genotype 78	-0.07	0.18	-6.99	0.00	-1.00	-2.50	0.45	0.15	2.40	0.07	1.00	1.50	-2.30	0.01	-0.02	3.06	-0.009
Genotype 79	-0.22	0.10	-7.45	3.50	1.00	-5.50	0.15	0.15	-26.85	-0.04	3.00	1.00	-1.00	0.01	-0.01	3.37	-0.028
Genotype 80	0.13	0.29	-7.10	2.00	0.50	-7.00	0.35	0.10	2.45	-0.03	1.50	0.00	-4.65	0.13	0.04	0.09	-0.018
Genotype 81	-0.12	0.20	-7.92	0.50	1.00	-3.25	0.25	0.10	-8.45	0.02	0.00	2.00	-0.05	0.12	-0.02	17.86	0.014
Genotype 82	-0.13	0.29	-13.83	0.50	0.50	-4.50	1.10	0.20	8.15	0.15	0.50	-1.00	-1.35	-0.03	-0.02	5.05	-0.007
Genotype 84	0.72	0.45	-5.42	1.00	0.00	-1.50	0.20	0.00	-19.40	0.02	1.00	2.00	2.75	0.13	0.06	-1.68	0.012
Genotype 85	-0.95	-0.24	-7.30	0.00	-0.50	-4.75	0.05	0.00	7.00	0.01	0.50	0.50	-1.35	0.04	0.01	8.04	0.002
Genotype 86	-0.75	-0.11	-8.49	1.00	1.00	-9.50	0.00	-0.10	10.10	-0.01	1.50	0.00	-1.50	0.11	0.03	0.83	-0.001
Genotype 87	0.63	0.42	-5.73	4.00	0.50	2.00	0.55	0.15	-4.45	0.03	4.00	2.50	-1.25	0.11	0.05	1.50	-0.017
Genotype 88	0.06	0.17	-5.37	5.00	3.00	-11.00	0.00	0.00	-17.75	-0.07	5.50	4.00	-2.75	0.26	0.12	-1.60	-0.016
Genotype 89	0.32	0.21	-3.45	1.50	0.00	-2.00	0.30	0.00	13.60	0.02	3.00	2.50	-1.50	0.13	0.05	-1.45	0.004
Genotype 90	0.75	0.42	-4.14	5.50	0.50	-4.25	0.20	0.00	14.85	-0.02	4.50	5.50	-2.25	-0.09	-0.02	-3.37	-0.032
Genotype 91	-0.94	-0.30	-9.27	0.50	0.50	-7.50	-0.10	0.00	-1.35	-0.02	1.00	0.50	-1.30	-0.26	-0.11	5.22	-0.060
Genotype 92	-0.27	-0.05	-1.79	3.00	6.00	-10.25	-0.45	-0.15	-12.95	-0.06	2.00	0.00	0.25	0.12	0.09	-16.40	0.002
Genotype 93	0.29	0.21	-2.82	0.50	-0.50	-5.65	-0.20	-0.05	-4.70	-0.06	1.00	-0.50	-2.65	-0.22	-0.05	-23.08	-0.073
Genotype 94	0.40	0.10	1.92	2.00	2.50	-4.05	0.35	0.20	-1.60	0.03	2.50	0.00	-1.50	-0.07	0.02	-14.17	-0.021
Genotype 95	-0.76	-0.18	-26.47	1.50	0.50	-7.50	-0.10	.	.	-0.04	0.00	3.00	-3.60	-0.05	.	.	-0.014
Genotype 96	-0.19	0.10	-6.40	2.00	3.00	-5.45	0.00	0.00	6.10	-0.01	2.00	2.50	-0.25	0.13	0.09	-18.37	0.006
Genotype 97	-0.71	-0.33	2.17	-1.00	0.50	-0.60	0.05	0.00	-3.05	0.02	0.00	0.00	0.65	-0.10	-0.03	-4.63	-0.018
Genotype 98	0.52	0.31	-5.11	0.50	-1.50	-4.75	-0.15	-0.05	-36.75	-0.02	1.50	1.50	-0.35	0.37	0.15	-8.83	0.042
Genotype 99	-1.54	-0.56	-3.36	0.50	0.00	-4.25	.	-0.05	-32.20	.	0.50	0.50	-2.75	0.01	0.00	3.44	-0.008
Genotype 100	0.20	0.08	1.25	3.00	2.00	-5.00	-0.20	-0.05	0.80	-0.09	2.50	3.50	-0.75	0.33	0.15	-12.56	0.037
Genotype 101	-0.24	0.15	-7.45	1.50	1.00	-1.85	0.10	0.10	-20.30	0.00	1.00	0.50	-0.90	-0.18	-0.03	-7.55	-0.032
Genotype 102	-0.69	-0.10	-9.76	2.00	2.50	-4.10	0.00	0.00	-31.90	-0.01	1.50	1.00	0.00	0.01	0.08	-32.95	-0.009
Genotype 103	-0.64	-0.07	-8.01	1.00	2.00	-0.30	-0.20	-0.05	-18.30	-0.04	0.50	1.00	0.00	0.27	0.10	-9.41	0.039
Genotype 104	-0.74	-0.05	-15.50	1.50	4.50	-7.00	0.05	0.05	-16.65	-0.01	0.50	0.50	-1.10	0.06	-0.05	10.12	0.004
Genotype 106	-0.53	-0.09	-5.37	0.50	1.00	-6.60	-0.20	-0.05	-7.40	-0.04	0.50	2.00	0.75	0.22	0.13	-15.66	0.032
Genotype 108	-0.20	0.10	-5.14	0.50	2.00	-3.15	-0.15	-0.05	-0.55	-0.03	1.00	-2.50	-2.25	0.07	0.01	0.18	0.000
Genotype 109	-0.84	-0.14	-11.25	-2.00	1.00	-4.35	-0.15	-0.05	-0.35	-0.01	-1.00	0.00	-0.50	0.03	0.01	-5.85	0.011

Genotype 110	-0.51	0.03	-6.92	1.50	1.00	-3.60	0.10	0.05	3.25	-0.01	0.50	0.50	6.00	0.43	0.20	-10.15	0.065
Genotype 111	0.18	0.27	-6.67	-1.00	0.00	-3.00	0.10	-0.05	13.35	0.03	-0.50	3.50	-3.00	0.34	0.11	4.46	0.051
Genotype 112	-0.04	0.12	-4.89	2.50	0.00	-10.00	0.45	0.10	1.95	0.03	3.50	1.50	-1.25	0.36	0.13	-3.96	0.017
Genotype 113	-0.30	-0.07	-2.48	0.50	-0.50	-7.00	0.30	0.00	13.15	0.03	0.00	3.50	2.15	0.16	0.06	0.70	0.021
Genotype 114	0.45	0.40	-6.98	1.00	-1.00	-2.50	0.65	0.10	3.20	0.06	2.50	2.50	-0.50	0.48	0.19	-4.62	0.033
Genotype 115	-0.73	-0.23	-4.89	-1.00	-1.00	-9.00	0.05	0.05	-6.00	0.01	0.50	-1.50	-2.50	0.11	0.02	0.20	0.010
Genotype 116	-0.10	0.09	-4.85	2.50	-0.50	-7.25	-0.15	0.05	-26.65	-0.03	2.50	2.00	-1.00	0.09	0.04	-0.74	-0.004
Genotype 117	0.30	0.28	-5.72	1.00	-2.00	-1.00	0.15	0.10	-0.15	0.01	0.00	3.00	1.15	0.09	0.07	-9.44	0.012
Genotype 118	-0.32	-0.05	-1.94	1.00	1.00	-5.50	0.35	0.05	7.70	0.04	2.50	1.50	-1.15	0.14	0.05	0.89	-0.008
Genotype 119	0.21	0.29	-7.76	2.50	0.50	-7.00	0.05	0.00	7.60	-0.02	3.00	3.00	-0.60	0.19	0.12	-22.35	0.001
Genotype 120	-0.76	-0.11	-9.00	1.00	1.00	-10.50	0.15	0.10	-7.85	0.01	1.00	0.50	-2.55	0.14	0.05	-0.45	0.015
Genotype 123	-0.77	-0.17	-4.31	-2.50	-2.00	0.50	0.20	0.10	-1.35	0.05	-2.50	0.00	-1.00	0.20	0.09	-5.57	0.036
Genotype 124	-0.22	0.11	-6.37	0.50	-0.50	-4.00	0.00	0.00	4.65	0.00	1.50	2.50	-2.20	-0.03	-0.02	3.90	-0.014
Genotype 125	-0.27	-0.09	-1.38	-2.50	-1.00	-3.00	-0.15	-0.05	-0.45	0.04	-1.00	-1.00	-0.75	-0.12	-0.03	-0.41	-0.007
Genotype 126	0.00	0.01	-1.07	1.50	1.50	-7.00	-0.20	0.00	-0.40	-0.10	2.00	0.50	-3.60	0.11	0.05	-4.13	-0.032
Genotype 127	-0.62	-0.33	-6.64	-1.00	-0.50	-3.50	-0.20	-0.05	-5.35	-0.02	-1.00	0.00	-3.00	0.02	-0.01	-1.55	0.009
Genotype 129	0.45	0.42	-8.11	1.00	-0.50	-4.00	-0.05	-0.05	-2.60	-0.01	1.00	1.50	1.00	-0.09	-0.07	11.25	-0.017
Genotype 130	-0.17	0.08	-4.42	1.00	0.00	-3.00	0.00	0.00	-0.90	-0.02	2.00	1.00	1.60	0.04	-0.01	8.67	-0.015
Genotype 131	-0.56	-0.10	-4.43	1.00	-1.50	-7.00	-0.75	-0.25	-9.65	-0.10	1.50	2.50	-1.90	0.17	0.05	2.98	0.008
Genotype 132	-0.11	0.15	-7.36	2.50	2.00	-2.00	-0.30	-0.05	-10.00	-0.10	3.50	2.50	0.25	0.15	0.07	-2.64	-0.020
Genotype 133	0.05	0.15	-3.45	5.00	2.50	-4.95	-0.10	0.00	-18.05	-0.04	3.50	3.00	1.00	-0.21	-0.04	-13.41	-0.036
Genotype 134	0.17	0.32	-9.18	1.50	2.00	-1.75	0.20	-0.10	23.70	-0.03	1.00	1.00	-0.90	-0.15	-0.05	-5.10	-0.081
Genotype 135	-0.28	-0.05	-3.57	-0.50	0.00	-0.30	0.10	0.05	-7.80	0.02	0.00	0.50	0.30	-0.49	.	.	-0.075
Genotype 137	-0.27	-0.02	-7.13	0.50	-2.50	1.90	0.10	0.05	-35.10	0.01	1.00	0.50	-0.50	-0.32	-0.08	.	-0.055
Genotype 138	-0.14	-0.02	-16.04	-1.50	0.00	-5.85	0.25	-0.05	4.45	0.16	-2.00	-1.00	-5.95	-0.37	-0.11	-44.68	-0.070
Genotype 140	0.19	0.36	-10.86	2.50	2.00	-7.65	0.10	0.05	-22.35	-0.01	3.00	0.00	-1.95	-0.47	-0.15	-31.74	-0.083
Genotype 141	0.13	0.25	-6.43	5.00	2.50	-3.55	0.25	0.20	-28.65	0.00	3.50	2.50	-2.25	-0.47	-0.18	-21.63	-0.059
Genotype 142	-0.19	0.07	-9.91	-2.50	-2.00	1.60	0.20	-0.05	11.05	0.06	-1.50	-1.50	0.25	-0.26	-0.12	17.72	-0.031
Genotype 143	-0.38	-0.02	-9.76	2.00	2.00	-6.75	0.00	0.00	-2.30	-0.01	1.50	1.50	-3.25	-0.22	-0.10	15.57	-0.042
Genotype 144	-0.65	-0.12	-11.33	1.00	1.50	-5.75	0.00	-0.05	-25.15	-0.01	0.00	1.00	-1.70	-0.57	-0.14	-31.89	-0.094
Genotype 145	-1.25	-0.24	-10.73	1.50	-3.00	-4.50	-0.05	0.05	-2.85	-0.01	1.50	1.00	0.25	-0.24	-0.06	-5.47	-0.034
Genotype 146	-0.55	0.08	-15.07	-2.00	-0.50	0.75	0.15	0.05	-7.90	0.04	-3.00	-2.50	2.25	-0.05	0.00	-7.19	0.009
Genotype 147	0.04	-0.09	2.44	-2.00	-2.50	-0.55	-0.05	0.05	-26.15	0.01	-3.00	-1.00	1.25	-0.10	-0.03	-1.81	0.001
Genotype 148	-0.74	-0.08	-7.75	0.50	1.00	-6.00	-0.35	-0.05	-27.65	-0.08	0.50	-1.00	-1.50	-0.07	-0.06	9.55	-0.019
Genotype 149	-0.09	0.14	-7.56	2.00	-0.50	-2.40	-0.10	0.05	-26.60	-0.03	1.50	1.50	0.10	-0.41	-0.19	10.80	-0.052
Genotype 150	-0.17	-0.25	7.24	2.00	3.00	-5.70	0.30	0.00	13.00	0.01	1.50	2.00	1.40	0.03	-0.01	5.15	-0.006
Genotype 151	-0.13	0.01	-0.35	1.50	2.50	-1.95	-0.05	-0.05	-3.45	-0.02	2.00	2.50	-2.90	0.11	0.01	12.14	0.010
Genotype 152	-0.24	-0.07	-1.65	-0.50	0.50	-2.15	-0.05	0.00	0.70	0.00	-1.00	-1.50	-2.70	-0.07	-0.01	-16.57	-0.002
Genotype 153	0.09	0.31	-12.59	2.00	2.00	2.40	0.50	0.25	-24.20	0.05	2.50	1.50	2.10	-0.12	0.00	-31.07	-0.040
Genotype 154	-0.15	-0.02	-24.61	2.50	2.00	-1.50	0.00	.	-3.60	-0.02	0.50	1.00	-2.90	0.00	0.00	-0.50	-0.005
Genotype 156	-0.70	0.10	-23.07	1.00	-1.50	-6.00	0.20	0.00	-12.95	0.02	3.00	-1.50	0.35	-0.21	-0.07	-11.84	-0.093
Genotype 157	-0.15	0.25	-18.16	-0.50	0.00	-1.00	0.45	0.05	-2.75	0.08	0.00	-1.00	-2.25	-0.27	-0.10	-3.33	-0.045
Genotype 158	0.42	0.44	-20.04	2.00	2.00	-5.00	-0.10	0.05	-27.45	-0.05	2.00	-0.50	4.00	0.10	0.08	-9.59	-0.017
Genotype 159	0.73	0.41	-14.34	2.50	1.50	-3.50	-0.05	.	.	-0.02	4.50	3.00	-1.40	0.03	0.11	-11.42	-0.051
Genotype 160	-0.29	0.16	-22.25	0.00	1.00	-3.50	-0.10	.	.	-0.02	0.50	-1.00	-2.00	0.06	0.02	-4.34	0.005
Genotype 163	-0.04	0.28	-13.05	0.50	0.00	-4.00	-0.15	-0.05	-7.55	-0.03	1.00	-1.00	-0.50	0.15	0.08	-12.33	0.015
Genotype 164	0.83	0.75	-14.86	0.50	1.00	-1.00	0.50	0.05	-1.50	0.09	1.00	0.50	0.00	-0.22	-0.30	22.87	-0.065
Genotype 166	-0.52	0.12	-12.45	-2.00	-2.00	-3.50	-0.10	-0.10	-2.20	0.00	-1.00	0.50	-0.55	0.14	0.07	-7.72	0.027
Genotype 167	-0.15	0.39	-15.60	1.00	1.00	-0.75	-0.05	-0.05	-6.95	-0.04	1.50	0.00	-3.35	-0.11	0.05	-19.55	-0.050

Genotype 168	1.00	0.89	-18.95	3.00	0.00	-1.50	0.20	0.05	-1.85	-0.01	1.50	0.00	-0.25	0.26	0.11	-7.70	0.022
Genotype 170	0.59	0.61	-10.31	3.00	2.00	-7.00	-0.40	-0.15	-1.75	-0.13	1.50	-0.50	-0.90	0.08	0.08	-11.60	-0.010
Genotype 171	0.79	0.65	-12.26	-1.00	-2.00	-7.25	0.35	0.05	10.90	0.10	0.00	1.50	0.60	-0.03	-0.02	6.28	-0.005
Genotype 173	0.54	0.64	-13.06	0.00	0.50	-3.50	0.20	-0.05	2.45	0.03	-0.50	-0.50	2.80	0.06	0.03	-1.80	0.014
Genotype 174	0.05	0.49	-16.54	-0.50	-3.50	-4.00	0.05	-0.10	16.05	0.01	1.50	0.00	2.00	0.17	0.09	-0.69	0.008
Genotype 175	0.59	0.59	-11.16	0.50	-1.50	-5.50	0.55	0.10	0.85	0.10	0.00	0.00	0.35	-0.09	-0.01	-14.31	-0.016
Genotype 176	-0.34	0.15	-9.69	1.00	0.50	-2.50	0.25	0.00	-2.55	0.03	2.00	2.00	-2.25	-0.10	-0.04	-0.64	-0.050
Genotype 177	0.56	0.70	-21.71	3.00	0.00	-6.50	-0.30	-0.10	-12.45	-0.08	1.50	1.50	-1.75	0.43	0.14	3.82	0.044
Genotype 178	-1.00	-0.16	-10.26	0.50	-1.50	-6.65	0.20	0.10	-7.35	0.02	-1.00	2.00	4.50	-0.06	-0.02	0.67	-0.001
Genotype 179	-0.32	0.11	-12.00	3.50	3.50	-1.35	0.05	0.00	-0.70	-0.01	4.00	2.00	0.75	-0.28	-0.12	10.58	-0.044
Genotype 180	-0.49	0.25	-13.16	1.00	0.00	-7.75	-0.20	-0.10	-6.45	-0.03	-1.00	0.00	4.00	0.20	0.08	-2.23	0.058
Genotype 181	0.22	0.22	-2.50	2.50	0.50	-0.55	0.35	0.20	-11.80	-0.05	5.50	4.00	2.50	0.16	0.00	13.71	-0.096
Genotype 182	0.15	0.28	-7.47	1.50	-0.50	-22.35	-0.30	-0.05	-8.30	-0.07	1.00	0.00	0.30	0.36	0.15	-6.82	0.049
Genotype 183	-0.04	0.26	-7.99	0.00	-1.00	-6.65	-0.05	0.05	-7.10	-0.01	1.00	2.00	-2.55	-0.28	-0.09	-0.69	-0.063
Genotype 184	-0.55	-0.10	-8.19	0.50	2.00	-3.70	-0.20	-0.05	-7.60	-0.05	1.00	0.50	-4.95	-0.25	-0.07	-7.11	-0.068
Genotype 185	0.28	0.26	-10.95	2.00	3.00	-1.50	0.05	0.00	-7.70	-0.01	1.50	-1.50	-2.60	-0.08	-0.04	8.98	-0.026
Genotype 187	0.11	0.16	-4.05	1.00	1.50	-3.75	0.00	0.05	-6.10	-0.01	2.00	1.50	-0.80	0.23	0.07	-0.77	0.015
Genotype 188	0.10	0.04	0.15	2.50	1.50	0.15	0.20	0.05	-6.30	0.00	2.50	2.00	0.15	-0.13	-0.06	5.22	-0.047
Genotype 189	-0.09	0.27	-8.28	-0.50	-0.50	-3.65	-0.20	0.00	-4.90	-0.02	0.50	0.00	-1.50	0.08	0.02	1.76	0.006
Genotype 190	0.49	0.41	-6.20	3.00	-1.50	-5.00	0.00	0.05	-7.60	-0.03	2.00	1.50	0.75	0.00	-0.02	5.25	-0.010
Genotype 191	0.22	0.23	-5.06	1.50	-0.50	1.35	0.00	-0.05	5.95	-0.04	2.00	1.00	-3.25	-0.06	-0.02	2.08	-0.043
Genotype 192	0.11	0.50	-11.19	0.50	0.00	-5.75	0.00	0.05	-5.10	-0.01	2.00	1.50	-0.75	-0.29	-0.10	4.39	-0.073
Genotype 193	0.29	0.41	-9.77	1.50	1.00	-4.25	-0.10	-0.10	1.45	-0.04	2.50	3.00	-0.25	0.22	0.08	-2.63	0.013
Genotype 194	0.08	0.11	-3.00	1.50	2.00	-1.80	-38.30	-0.10	-16.60	-6.99	1.50	3.50	-2.00	-0.23	-0.10	5.33	-0.048
Genotype 195	0.14	0.19	-4.46	-0.50	2.00	-8.25	-0.20	0.00	-5.10	-0.03	1.50	0.50	0.15	0.20	0.02	9.88	0.014
Genotype 198	0.35	0.34	-8.83	0.00	1.00	-3.10	-0.25	-0.15	6.05	-0.04	0.00	2.50	0.75	0.10	0.02	4.35	0.015
Genotype 199	0.34	0.16	-1.45	4.00	3.50	-3.30	-0.10	.	-8.50	-0.03	1.00	1.50	-2.25	0.01	-0.03	16.34	-0.003
Genotype 200	-0.01	0.10	-5.37	-3.00	1.00	-2.75	-0.15	0.00	-6.85	-0.01	3.00	1.00	-1.00	0.06	0.00	1.96	-0.010
Genotype 201	0.36	0.40	-11.77	3.50	-1.50	-8.50	0.10	-0.10	7.80	-0.01	4.50	5.00	-1.00	0.00	0.00	2.97	-0.011
Genotype 203	0.46	0.48	-16.89	3.50	2.00	-0.25	0.25	-0.05	9.45	0.00	3.50	2.00	0.30	0.11	0.05	-0.93	-0.004
Genotype 205	-0.33	-0.04	-5.80	0.00	-2.50	-2.75	0.20	0.00	9.00	0.03	0.00	1.00	-0.35	0.06	0.04	-5.58	0.009
Genotype 206	-0.62	-0.15	-6.82	-0.50	1.00	-3.00	0.15	0.00	3.20	0.05	0.00	1.00	-1.35	0.06	0.00	8.11	0.016
Genotype 207	0.60	0.42	-11.97	2.50	2.50	-1.25	0.30	0.10	0.15	0.02	4.50	2.50	0.50	-0.02	-0.01	11.30	-0.021
Genotype 208	-0.38	0.14	-15.46	0.00	-1.00	-5.50	0.00	-0.15	16.00	0.00	2.00	3.50	0.00	0.04	0.02	-0.33	-0.006
Genotype 209	-0.13	0.21	-12.80	-1.00	-1.00	-5.00	0.30	0.00	2.65	0.11	0.00	0.50	3.30	0.02	0.00	0.63	0.004
Genotype 211	2.16	0.96	3.22	0.00	0.00	1.75	0.95	0.25	6.85	0.27	0.00	0.50	3.85	0.40	-0.01	11.63	0.100
Genotype 212	-0.35	0.45	-7.09	1.50	1.00	-6.50	0.35	0.05	0.15	0.03	1.50	4.50	0.00	0.07	0.02	-2.49	-0.004
Genotype 213	0.67	0.55	-12.00	-1.50	0.50	-4.00	0.20	0.05	1.00	0.03	1.50	3.00	2.75	0.21	0.10	-3.96	0.014
Genotype 214	-0.10	0.24	-14.23	-2.50	3.00	-4.50	0.30	0.10	-5.50	0.05	4.50	2.50	1.40	0.12	0.05	2.26	-0.013
Genotype 215	-0.39	0.13	-10.06	-1.00	-1.00	-5.75	0.25	0.05	-3.10	0.07	-0.50	-3.00	-0.90	0.01	0.03	-3.53	0.009
Genotype 216	0.34	0.45	-10.81	0.50	-0.50	-3.00	0.45	0.05	3.85	0.08	0.50	0.00	2.35	0.05	0.04	-0.27	-0.004

BFW- before cut back fresh weight; BDW- before cut back dry weight; BMST- before cut back moisture content; ATN- after cut back tiller number; ANL- after cut back new leaf; APHT- after cut back plant height; AFW- after cut back fresh weight; ADW- after cut back dry weight; AATW- after cut back average tiller weight; AMST- after cut back moisture content; RTN- return to normal growth tiller number; RNL- return to normal growth new leaf; RPHT- return to normal growth plant height; RFW- return to normal growth fresh weight; RDW- return to normal growth dry weight; RATW- return to normal growth average tiller weight; RMST- return to normal growth moisture content.
